# The biochemical composition and transcriptome of cotyledons from *Brassica napus* lines expressing the AtGL3 transcription factor and exhibiting reduced flea beetle feeding

**DOI:** 10.1186/s12870-018-1277-6

**Published:** 2018-04-16

**Authors:** Margaret Gruber, Ushan Alahakoon, Ali Taheri, Nayidu Nagubushana, Rong Zhou, Banyar Aung, Andrew Sharpe, Abdelali Hannoufa, Peta Bonham-Smith, Dwayne D. Hegedus D

**Affiliations:** 1 Saskatoon Research Centre, Agriculture and Agri-Food Canada, 107 Science Place, Saskatoon, SK S7N0X2 Canada; 2 Department of Biology, University of Saskatchewan, Saskatoon, SK Canada; 3 Present Address: DOW Agro-Sciences, Saskatoon, SK Canada; 4 Present Address: Department of Agricultural and Environmental Sciences, Tennessee State University, Nashville, TN USA; 5 Global Institute for Food Security, University of Saskatchewan, Saskatoon, SK Canada; 6 Agriculture and Agri-Food Canada, London, ON Canada; 7 Department of Biology, Western University, London, ON Canada; 8 Department of Food and Bio-Product Sciences, University of Saskatchewan, Saskatoon, SK Canada

**Keywords:** *Brassica napus*, Trichomes, Cotyledons, Glucosinolates, Flea beetle, RNA sequencing

## Abstract

**Background:**

Previously, transgenic trichome-bearing (hairy leaf) *Brassica napus* lines expressing either the *Arabidopsis thaliana GL3* gene (line AtGL3+) [1] or the *AtGL3* gene in combination with an RNAi construct to down-regulate *TTG1* (line K-5-8) [2] were developed. The leaves of these lines exhibited altered insect feeding (flea beetle) and oviposition (diamondback moth) behaviour compared to the non-transgenic semi-glabrous leaves of *B. napus* cv. Westar. Interestingly, the cotyledons of these lines remained glabrous, but also showed reduced feeding by flea beetles. Here we examine the composition and global transcriptome of the glabrous cotyledons from these transgenic lines to ascertain the mechanism(s) underlying this unexpected phenomenon.

**Results:**

Approximately, 7500 genes were up-regulated in cotyledons of each hairy line, compared with < 30 that were down-regulated. The up-regulated genes included those involved in cell wall synthesis, secondary metabolite production, redox, stress and hormone-related responses that have the potential to impact host plant cues required to elicit defense responses toward insect pests. In particular, the expression of glucosinolate biosynthetic and degradation genes were substantially altered in the glabrous cotyledons of the two hairy leaf lines. The transcriptomic data was supported by glucosinolate and cell wall composition profiles of the cotyledons. Changes in gene expression were much more extreme in the AtGL3+ line compared with the K-5-8 line in terms of diversity and intensity.

**Conclusions:**

The study provides a roadmap for the isolation and identification of insect resistance compounds and proteins in the glabrous cotyledons of these hairy leaf lines. It also confirms the impact of mis-expression of *GL3* and *TTG1* on types of metabolism other than those associated with trichomes. Finally, the large number of up-regulated genes encoding heat shock proteins, PR proteins, protease inhibitors, glucosinolate synthesis/breakdown factors, abiotic stress factors, redox proteins, transcription factors, and proteins required for auxin metabolism also suggest that these cotyledons are now primed for resistance to other forms of biotic and abiotic stress.

**Electronic supplementary material:**

The online version of this article (10.1186/s12870-018-1277-6) contains supplementary material, which is available to authorized users.

## Background

Plants possess a variety of biochemical and morphological defences aimed at deterring insects from herbivory and oviposition. Defences can be either induced upon attack and localized to the site of attack, be systemic, or both (reviewed in [3]). Host plant resistance is categorized into three groups: i) antibiosis, resulting in increased mortality, reduced longevity or reduced reproduction of the insect; ii) antixenosis, affecting the behaviour of the insect and often expressed as non-preference for a resistant plant compared to a susceptible plant; and iii) tolerance, this being the ability of a plant to withstand or recover from insect damage and perform better than a susceptible plant grown under similar conditions [4]. Crops with insect resistance can reduce the accumulation of harmful chemical residues in the environment, as well as provide economic benefits to farmers and seed producers.

Glabrous (smooth) and semi-glabrous (few trichomes) lines of *Brassica napus* L. and *Brassica rapa* L. canola (oilseed rape) and Brassica vegetable crops are susceptible to many specialist and generalist insect pests. Flea beetles (FBs) [*Phyllotreta cruciferae* (Goeze) and *Phyllotreta striolata* (Fab.)] are specialist *Brassica* pests in several parts of the world, including Canada [5], India, and Eastern Europe [6, 7]. FBs attack at the crucial seedling stage as well as at more mature stages, such as leaves and developing green pods, where they reduce seed yield and grade. FB have developed resistance to insecticides used for their control (reviewed in [8, 9]). This highlights the importance of developing insect-resistant cultivars to reduce the use of chemical protection methods.

Trichomes (leaf hairs) have evolved as a physical defence against herbivore feeding and oviposition [10], and trichome density and length both negatively impact these processes in many insect species. Trichomes on *Arabidopsis thaliana* L. develop at the distal end of the developing leaf, thereby protecting the more supple, younger parts of the leaf that are preferred for diamondback moth oviposition and more vulnerable to FB and *Psylloides* sp. feeding damage [11, 12]. Mature *Brassica villosa* leaves have an extremely high density of trichomes (~ 4000 cm^− 1^) and are immune to FB damage in the field as the insects avoid the leaves [13].

Several *Arabidopsis thaliana glabrous* (lacking trichomes) mutants are also deficient in the production of secondary metabolites, most notably anthocyanins. The link between trichome formation and secondary metabolite production occurs through sharing of components within the Myb/bHLH/TTG1 (MBW) transcriptional regulatory complex. In trichome formation, the basic helix-loop-helix transcription factors GL3, or the similar factor ENHANCER OF GL3, form a regulatory complex with the R2R3-MYB factor GL1 and the WD-repeat protein TTG1 which interacts with GL3 and EGL3. This regulatory complex activates the expression of genes encoding a secondary set of transcription factors comprising GL2, TTG2 and SIM to induce trichome formation. In addition, at least 6 other R3 MYB proteins (CPC, TRY, ETC1, ETC2, ETC3 and TCL1) can replace GL1 to disrupt and/or alter the specificity of the regulatory complex [14–16]. Genes encoding enzymes involved in the later stages of anthocyanin biosynthesis are regulated by a similar complex consisting of TTG1 and GL3, but with different MYB factors [17].

Earlier, we demonstrated that ectopic expression of *A. thaliana GL3* dramatically increases trichome formation on *B. napus* leaves, but compromises plant development [1]. Trichome production is further enhanced, while the developmental abnormalities are alleviated, when the expression of the endogenous *TTG1* gene is reduced through the introduction of an RNAi construct [2]. Like hairy *B. villosa*, FB feeding is reduced on seedlings of the hairy leaf transgenic *B. napus* lines [1, 2, 18] as the insects do not initiate feeding probes on the trichome-enhanced leaves [2, 19]. The leaves of these lines exhibit large changes to their transcriptomes and growth patterns [2]. Curiously, the glabrous cotyledons from the hairy-leaf transgenic *B. napus* lines also showed highly reduced FB feeding (30–50%) [18]. It was suggested that the antixenotic effect could be due to altered plant architecture as the cotyledons in both transgenic lines are initially vertically oriented before becoming horizontally oriented as the plant develops. However, they could not rule out the possibility that a change in biochemical composition was also involved. Attraction and stimulation of feeding in FBs is governed principally by glucosinolates and/or their breakdown products from the host plant [20]. This suggested that the composition and gene expression patterns of the *AtGL3* transgenic glabrous cotyledons may also have been altered so as to impact FB behaviour. Here, we examine the composition of glabrous cotyledons of the two transgenic hairy *B. napus* lines: one hairy leaf line expressing the *AtGL3* gene (AtGL3+) and one ultra-hairy leaf line (K-5-8) expressing *AtGL3* and down-regulated in the expression of the *BnTTG1* gene. We demonstrate that the glabrous cotyledons of these lines exhibited altered secondary metabolite (anthocyanins and glucosinolates) and lignin content, as well as altered expression of genes specifying secondary metabolite biosynthesis and degradation, cell wall biosynthesis, hormones, and redox proteins which may have contributed to changes in host plant cues for insect pests.

## Methods

### Plant material

Untreated seeds of three plant entries were used in this study: the semi-glabrous leaf *B. napus* cv. Westar (parent line), a homozygous hairy-leaf *AtGL3* transgenic *B. napus* developed from a cv. Westar parental background (line AtGL3+, 1), and a homozygous ultra-hairy transgenic leaf *B. napus* (line K-5-8) having highly reduced *BnTTG1* expression within the *AtGL3*+ *B. napus* background [2]. Seeds were sterilized and placed onto solid MS media. Cotyledons for glucosinolates (GS), cell wall component analysis, and RNA sequencing were harvested from 7-day old seedlings grown in three replicates in magenta jars (10 seeds/replicate) under insecticide-free conditions in a controlled plant growth chamber (22/18^o^ C; 16 h photoperiod; 60–80 μmoles.m^− 2^.s^− 1^). Cotyledons for qRT-PCR of trichome genes were grown at 22/24^o^ C under a 16 h photoperiod with light at 400 μE. m^− 2^.s^− 1^. Cotyledons used to measure phenotype changes, anthocyanin content, and qRT-PCR of anthocyanin genes were grown for 10 days under continuous light (400 μE. m^− 2^.s^− 1^).

### Cotyledon composition analysis

Seven-day-old cotyledons were extracted and glucosinolates (GS) converted to desulfoglucosinolates (DS-GS) based on the AOCS Official Method Ak 1–92. Specifically, freeze-dried cotyledons (~ 0.1 g) were agitated with steel rods (25 × 8 mm) on an Eberbach reciprocating shaker for 10 min at 280 rpm, and then 3 ml of methanol and 1 ml of 0.2 mM benzyl GS were added and shaking continued for 60 min. After centrifugation at 2300 g for 15 min, 3 ml of supernatant was loaded onto 0.3 ml pre-swollen DEAE-Sephadex resin (~ 30 mg) in Bio-Spin micro-columns (Bio-Rad, Mississauga Canada). The resin was rinsed with 1.5 ml of 2% acetic acid, 1.8 ml of water and 1.2 ml of 20 mM sodium acetate at pH 4.0, and then 100 μl of a purified sulfatase solution was added to the resin and the micro-columns sealed and incubated at 20 °C overnight. DS-GS were eluted with 1.2 ml of water, filtered, and separated using a Waters UPLC-PDA-TQD system and a BEH Shield RP18 column (2.1 × 50 mm; 1.7 μm) (100% water at 0.8 mL/min) for 0.3 min, followed by a linear gradient of 0% to 25% acetonitrile (*v*/v) over 6.7 min. The DS-GS were quantified at 229 nm and identified by monitoring the characteristic loss of 162.2 mass units using MS/MS constant neutral loss scans.

Cell wall carbohydrates and lignin were measured on purified cell wall residue (CWR) as per methods in UpdeGraff [21], Brinkman et al. [22], and Aung et al. [23]. CWR was extracted in phosphate buffer with Triton X-100 using 10 mg of seven-day-old cotyledons. Lignin content was analysed on CWR based on the thioglycolate-alkaline hydrolysis assay [22] and quantified using UV/VIS spectrophotometry at 280 nm and a calibration curve developed with commercial lignin (Sigma-Aldrich, Oakville, ON, Canada). Total acid-releasable cellulosic glucose was determined from CWR using a commercial cellulose standard (Sigma-Aldrich) and anthrone reagent spectrophotometry at 280 nm after sugar and starch removal based on the method of Theander et al. [24].

### Cotyledon RNA sequencing

Total RNA was extracted from cotyledons of seven-day-old seedlings using an RNAeasy Mini Kit with contaminating gDNA being removed using RNAse-free DNAse™ (Qiagene Inc., ON Canada). RNA samples were quantified and RNA integrity determined using an RNA6000 nano assay in an Agilent 2100 Bioanalyzer™ (Agilent Technologies, Palo Alto, CA USA). RNA library preparation and sequencing were carried out using the Illumina TrueSeq RNA sample preparation platform v.2 with multiplex labeling following the manufacturer’s protocols. Details on cDNA library development, RNA sequencing, and data analysis were identical to those found in Alahakoon et al. [2]. Cotyledon gene expression changes in the two transgenic hairy leaf lines relative to cv. Westar cotyledon expression were then introduced into MAPMAN for allocation into 36 functional barcode index numbered (BIN) categories [25].

### Quantitative-reverse transcription PCR (qRT-PCR) analysis

qRT-PCR was conducted on 10-day-old cotyledons to test *AtGL3* expression and the summed transcripts of all homeologues for each of five *B. napus* regulatory genes (*BnGL1*, *BnGL2*, *BnGL3*, *BnTTG1* and *BnTRY*) encoding proteins known to be involved in the trichome MYB-*β*HLH − WD40 tri-protein initiation complex as outlined in Alahakoon et al. [2].

### Statistical analysis

Cotyledon composition and qRT-PCR data were analyzed with either one-way or two-way ANOVA using a MIXED model in SAS 9.2 [26] or a t-test. Assumptions of ANOVA were tested using a Normality test (Shapiro-Wilk), an Equal variance test (Brown Forsythe) and a Levenes test. Means were compared using a Tukey test or pairwise using a Dunn’s method in SAS 9.2, and treatments were declared significant at *P* ≤ 0.05 and trends declared at *P* ≤ 0.1. Read counting and statistical analysis of the RNA-seq data were carried out using Cuffdiff in the Cufflinks software package [27].

## Results

### Cotyledon composition

**Fig. 1:**
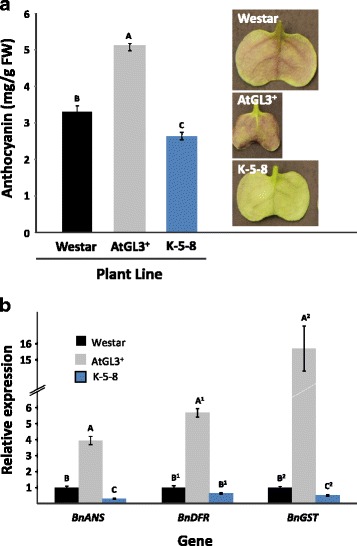
Total seedling anthocyanins and qRT-PCR of anthocyanin gene expression in *B. napus* cv. Westar and transgenic lines (AtGL3+ and K-5-8) grown under 24 h continuous light. Panel A: Anthocyanins. Insert shows colour and morphology on the abaxial surface of the cotyledon. Panel B: qRT-PCR of anthocyanin genes. Expression of individual genes is relative to that of glabrous *B. napus* cv. Westar (set at 1), which has been normalized to the expression of the *B. napus Act2* gene. A Tukey test was used to detect significant differences in total anthocyanins or expressed genes between the plant lines. Means (*n* = 3) + standard error with different letters differ significantly (*p* ≤ 0.05). FW = fresh weight

As with true leaves, the glabrous AtGL3+ cotyledons were much smaller than Westar and K-5-8 cotyledons (Fig. 1 insert), reflecting the smaller stature and lower vigor of the AtGL3+ line, while the K-5-8 line produced much larger cotyledons and subsequently plants with higher vigour [2, 18]. We compared the biochemical composition of the cotyledons expressing *AtGL3* to that of the wild-type line to determine if particular specific secondary metabolites or polymers contributing to cell fortification could be correlated with altered insect behaviour. Anthocyanins are particularly useful in young plants as a means of insect defense as they alter the spectral properties of the plant, potentially making it less visible, and increase their phenolic content [28]. AtGL3+ cotyledons also exhibited more red coloration on the abaxial surface than Westar cotyledons, while the K-5-8 cotyledon abaxial surface was green when grown indoors under continuous light to stress the cotyledons (Fig. 1a insert). The red coloration was due to increased anthocyanin (Fig. 1a) and an increase in transcription of three anthocyanin genes (*BnANS*, *BnDFR* and *BnGST*) in AtGL3+ cotyledons was noted compared to the other two lines (Fig. 1b).

**Fig. 2:**
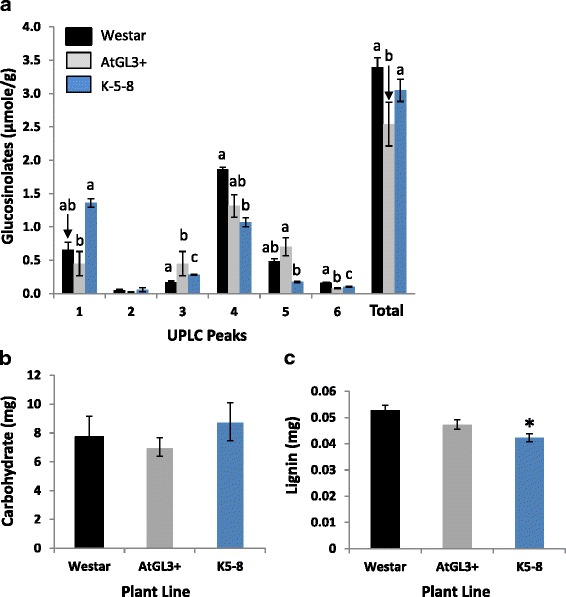
Composition of 7-day-old glabrous cotyledons. **a** Glucosinolates. UPLC peaks represent: 1, progoitrin (2-OH-3-butenyl-GS); 2, glucoraphanin (4-methylsulfinylbutyl-GS); 3, 3-butenyl-GS; 4, 4-hydroxy-3-indolylmethyl-GS; 5, 4-methoxy-3-indolylmethyl-GS; 6, 4-methoxy-glucobrassicin (4-methoxy-indol-3-ylmethyl-GS). Different letters indicate pairwise significance difference of the means (± SD) for each line within each compound type (*p* < 0.05). **b** Cell wall residue carbohydrates. **c** Cell wall residue lignin. Asterisk (*) indicates significance difference of the means (± SD) relative to cv. Wester (*p* < 0.05)

Glucosinolates are sulfur-linked glucosides commonly found in species within the Brassicaceae/Cruciferae [29]. Feeding initiation in crucifer specialists, such as FB, are influenced by glucosinolates and their breakdown products derived from the host plant [20]. The level of GSs in glabrous cotyledons of the two transgenic lines and Westar was 1000-fold lower than GS levels in the seed (data not shown). Total GS levels in K-5-8 cotyledons were not significantly different from those of Westar, while total GS in the AtGL3+ line was lower (Fig. 2a). The GS profile of the lines expressing *AtGL3* was also found to be different from that of Westar and different from one another. Progoitrin (2-OH-3-butenyl-GS), 4-hydroxyglucobrassicin (4-hydroxy-3-indolylmethyl-GS) and glucobrassicin (indol-3-ylmethyl-GS) were the most abundant cotyledon GSs. While progoitrin levels in the transgenic lines were similar to Westar, the level was significantly higher in the K-5-8 line when compared to AtGL3+. The level of glucoraphanin (4-methylsulfinylbutyl-GS) was not significantly different among the lines, whereas the level of gluconapin (3-butenyl-GS) was significantly higher in the transgenic lines and further elevated in the AtGL3+ line. 4-hydroxyglucobrassicin levels were reduced with the K-5-8 lines having only 60% of that in Westar. Glucobrassicin levels in the transgenic lines were statistically similar to Westar; however, the K-5-8 cotyledons had significantly less of this GS than those of AtGL3+. Both types of transgenic cotyledons had lower 4-methoxy-glucobrassicin (4-methoxy-indol-3-ylmethyl-GS) levels compared to Westar with the AtGL3+ having even lower levels than the K-5-8 line.

Changes in the composition of the plant cell wall (e.g. cellulose, hemicellulose, pectin and lignin) serves to fortify plant tissues and can make them more difficult to consume and more difficult from which to extract nutrition [30]. Cotyledon cell wall carbohydrate was similar between the two transgenic lines and cv. Westar (Fig. 2b). Lignin extracted from the cell wall residue was similar between Westar and the AtGL3+ line, but was significantly reduced in K-5-8 cotyledons (Fig. 2c).

### Cotyledon transcriptomes related to insect host cues, metabolism and regulation

**Fig. 3:**
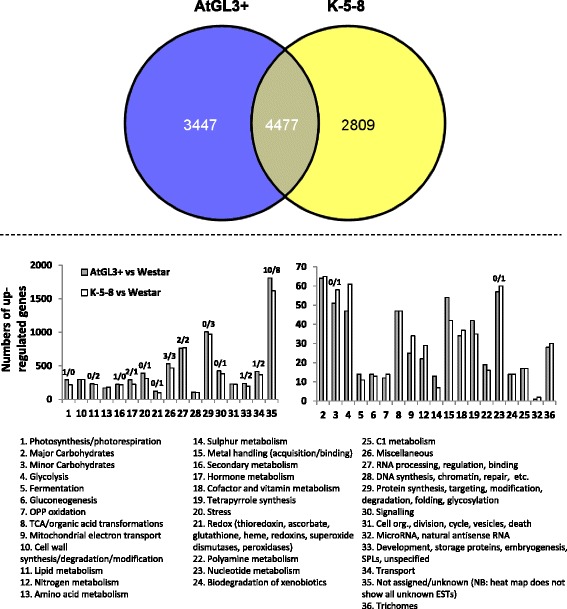
Overview of changes in glabrous cotyledon gene expression in 7-day-old hairy leaf (AtGL3+) and ultra-hairy leaf (K-5-8) *B. napus* lines relative to cv. Westar. Upper Panel: Venn diagram showing the number of up-regulated genes. Lower Panel: Mapman categories. The graphs show different data graphed on expanded or smaller Y axes. #/# above some pairs of bars indicate the number of down-regulated genes in that bin

The glabrous cotyledons of the K-5-8 and AtGL3+ lines were as resistant to FB feeding as those from pesticide-treated Westar [18]. To further explore these phenomena, we conducted RNA-Seq analysis to identify transcription patterns in cotyledons which might affect insect behaviour. Cotyledons of the AtGL3+ line and K-5-8 showed up-regulation of 7924 and 7286 genes relative to Westar cotyledons, respectively, of which 4477 were common (Fig. 3). Curiously, fewer than 30 genes were down-regulated in either the hairy line or the ultra-hairy line relative to Westar (Fig. 3; Additional file  3: Figure S1 and Additional file 4: Figure S2). These genes could be organized within 36 MAPMAN functional categories (BINS), with the largest number of up-regulated genes falling within 17 functional categories (Fig. 3; Additional file 1: Table S1). Categories with moderate (ca. 20–200) numbers of up-regulated genes relative to Westar cotyledons, included those specifying photosynthesis (BIN #1), carbohydrates (BINs #2, 3), glycolysis (BIN #4), TCA/organic acid transformation (BIN #8), cell wall synthesis (BIN #10), lipid and amino acid metabolism (BIN #11, 13), metal handling (BIN #15), secondary metabolism (BIN #16), hormones (BIN #17), stress (BIN #20), redox (BIN #21), nucleotide-related (BIN #23), DNA-related (BIN #28), signalling (BIN #30), cell organization, etc. (BIN #31), development (BIN #31), and transport (BIN #34) (Fig. 3; Additional file 3: Figure S1 and Additional file  4: Figure S2). Categories with a large number of up-regulated genes (> 500) included those without known functions (BIN #26), RNA-related (BIN #27), protein-related (BIN #29) and miscellaneous functions (BIN #35; e.g. cytochrome P450 enzymes, carbohydrases, lipases) (Fig. 3). Moreover, ~ 25% of all genes that exhibited different expression patterns in the transgenic cotyledons could not be assigned a function (BIN #35).

**Fig. 4:**
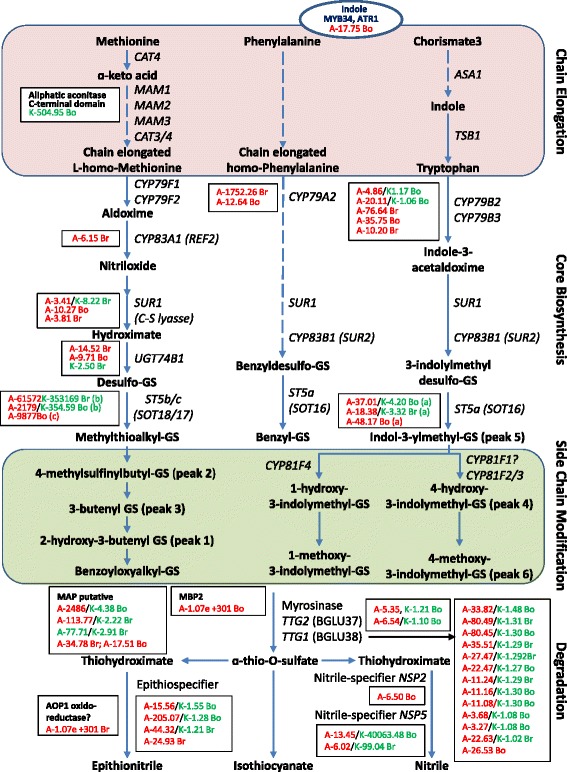
Changes in expression of genes related to glucosinolate biosynthesis and degradation in 7-day-old cotyledons grown under a 16/8 h diurnal light cycle. Figure shows GS UPLC peaks and gene expression changes (up-regulation) for the AtGL3+ line (A) and the K-5-8 line (K), each relative to *B. napus* cv. Westar levels. Br, *Brassica rapa* A-genome homeologue. Bo, *Brassica oleracea* C-genome homeologue. Gene IDs and expression levels can be viewed in Table 2

Within the functional categories above, a suite of genes potentially involved in insect resistance and specifying tissue toughness (wax, cell wall carbohydrates/proteins and lignin synthesis), metal handling, flavonoid glycosylation, as well as phenylpropanoid, alkaloid, and cyanogenic glycoside synthesis, were strongly up-regulated in glabrous cotyledons of both the AtGL3+ hairy leaf line and the K-5-8 ultra-hairy leaf line relative to Westar cotyledons (Table 1, Additional file 1: Table S1; Additional file 5: Figure S3). Genes involved in GS biosynthesis and degradation were also up-regulated in both types of transgenic cotyledons, but a much larger number of these genes tended to be represented in transcriptomes of AtGL3+ cotyledons than in K-5-8 cotyledons (Fig. 4; Additional file 2: Table S2). In particular, genes encoding proteins involved in core biosynthesis of methionine-based, indole and benzyl GSs, including cytochrome P450s and a sulfotransferase (*SOT18*), as well as a MYB factor, were > 2-fold up-regulated in the AtGL3+ line, although genes encoding *SOT18* and an aliphatic acconitase were highly up-regulated in the K-5-8 line (Fig. 4; Additional file 1: Table S1 and Additional file 2: Table S2). Moreover, a large number of genes involved in GS degradation, including those encoding myrosinase, a number of myrosinase-associated proteins (MAP), a myrosinase binding protein (MBP), an AOP1 oxidoreductase, and nitrile-specifier and epithio-specifier proteins were also up-regulated more strongly in the AtGL3+ line (Fig. 4). Finally, a large number of stress response genes were up-regulated in both types of transgenic cotyledons, including those responsive to biotic, drought and salt stress, those encoding pathogenesis-related (PR) proteins, PR-related protease inhibitors, heat shock proteins, and redox proteins known to assist with protection against reactive oxygen species (ROS), as well as those involved in hormonal control of growth and development (Additional file 2: Table S2; Additional file 6: Figure S4 and Additional file 1: Table S1). Genes encoding a wide variety of metabolism and transcription factors, or enzymes involved in protein modification and degradation, were strongly up-regulated in both AtGL3+ and K-5-8 cotyledons (Additional file 6: Figure S4 and Additional file 7: Figure S5).

**Table 1:** Cotyledon genes (common to two hairy lines) with potential to impact host plant responses to flea beetles and diamondback moth

			Fold change relative to Westar	
Gene ID	Sub-categories	Description	AtGL3 line	K-5-8 line^a^
Metal Handling				
bo7g039050	general	SBP3 (selenium-binding protein 3)	1.07E+301	1.07E+301
bo9g123330	binding, chelation, storage	NAS2 (NICOTIANAMINE SYNTHASE 2)	3.96E+08	3.92E+09
bo4g040020	"	ATSERAT2;1, SAT5, SAT1 (O-SERINE ACETYLTRANSFERASE 2;1)	8.99	352.70
bra002851	"	NAS2 | NAS2 (NICOTIANAMINE SYNTHASE 2)	47.80	77.58
bra015594	"	MT1C; copper ion binding	4.42	10.20
bra009595	"	MT2A, ATMT-K, ATMT-1 (METALLOTHIONEIN 2A)	3.23	7.38
bo5g008330	"	MT1C; copper ion binding	4.41	7.17
Wax				
bo8g102710	synthesis	KCS4 (3-KETOACYL-COA SYNTHASE 4)	3.33	5.83
bra033983	"	YBR159, KCR1 | YBR159; ketoreductase/oxidoreductase	4.59	2.81
bra004034	"	CUT1, POP1, CER6, G2, KCS6 (3-KETOACYL-COA SYNTHASE 6)	29.07	2.58
bo7g019710	"	KCS9 (3-KETOACYL-COA SYNTHASE 9)	1.67	2.42
bra024749	"	CUT1, POP1, CER6, G2, KCS6 (3-KETOACYL-COA SYNTHASE 6)	2.83	1.51
Cell Wall				
bra011899	modification	ATEXLA2, EXPL2, ATHEXP BETA 2.2 (EXPANSIN-LIKE A2)	9.17	12.64
bra024848	"	EXGT-A3, XTH27, hydrolase, xyloglucan:xyloglucosyl transferase	3.29	4.03
bo3g018260	precursor synthesis	UXS3 (UDP-GLUCURONIC ACID DECARBOXYLASE 3)	4.72	18.03
bra006722	"	"	3.87	9.62
bra021798	"	ATCSLB03, ATCSLB3 cellulose synthase/ glycosyl transferase	6.43	5.18
bo1g037390	"	ATCSLG2 cellulose synthase/ glycosyl transferase	3.73	3.17
bra016440	cell wall proteins	unknown protein	8.59	101.30
bo4g194100	"	proline-rich extensin-like family protein	5.50E+04	11.24
bra037731	"	"	33328.67	7.21
bra026268	"	hydroxyproline-rich glycoprotein family protein	20.79	5.06
bo1g051380	"	"	9.65	4.97
bra003200	degradation.mannan-xylose-arabinose-fucose	PMR6 (powdery mildew resistant 6); pectate lyase	5.41E+12	190.90
bra024089	"	MERI5B, MERI-5, SEN4 (meristem-5); hydrolyzing glycosyl bonds	3.13	66.59
bo4g108130	"	pectinesterase family protein	6.87	43.88
bra009234	"	ATPGIP1 (POLYGALACTURONASE INHIBITING PROTEIN 1); binding	44.74	37.33
bo2g127040	"	pectinacetylesterase, putative	3.30	19.53
bra010038	"	ATBXL1 (BETA-XYLOSIDASE 1); hydrolyzing O-glycosyl compounds	1.70	9.03
bo2g013480	degradation.pectate lyases/polygalacturonases	glycoside hydrolase family 28 protein	5.78	7.76
bo6g058470	"	PMR6 (powdery mildew resistant 6); pectate lyase	1.96E+05	4.92
Lignin				
bo3g024650	biosynthesis	ATC4H, C4H, CYP73A5 (CINNAMATE-4-HYDROXYLASE)	2717.78	39.35
bra036480	"	HCT (SHIKIMATE/QUINATE HYDROXYCINNAMOYL COA-TRANSFERASE)	2.72	2.68
Flavonoids				
bra018364	dihydroflavonols	UGT71D1 (UDP-GLUCOSYL TRANSFERASE 71D1)	7.75	12.33
bra037386	"	"	7.15	9.32
bo9g003740	"	"	65.69	6.15
Phenylpropanoids				
bra028893	phenylpropanoids	transferase family protein	14.84	17.57
bra029364	"	NIC2 (NICOTINAMIDASE 2); catalytic nicotinamidase	5.46	8.84
bo3g167180	"	O-methyltransferase family 2 protein	10.07	8.41
bo7g064020	"	transferase family protein	12.06	4.93
Alkaloids				
bra003263	N misc. alkaloid-like	strictosidine synthase family protein	416.91	3.94
bo6g054230	"	"	17.29	2.92
Cyanogenic glucosides				
bra014956	cyanase	CYN (CYANASE); DNA binding / cyanate hydratase/ hydro-lyase	7.06	6.21

^a^Cotyledon values are arranged from highest expression to lowest expression within each functional category using the K-5-8 line.

In general, more genes in each category were upregulated in the AtGL3+ line than in the K-5-8 line relative to Westar, for example, in hormones (BIN #17), stress (BIN #20), miscellaneous (BIN #26), protein (BIN #29), signalling (BIN #30), development (BIN #33), and transport (BIN #34) (Fig. 3; Additional file 1: Table S1). Up-regulated genes that were common to both hairy leaf lines often exhibited a greater level of expression in the AtGL3+ line than in the K-5-8 line (Tables 1, 2, and 3). This included genes involved in cell wall synthesis (cell wall proteins, pectate lyases, and lignin synthesis), and stress-response genes encoding chlorophyllase (COR1), PR protease inhibitors, and a *SENESCENCE ASSOCIATED GENE* 20, while the same genes were only moderately up-regulated within K-5-8 cotyledons. Up-regulated genes that were unique to AtGL3+ and K-5-8 cotyledons were linked to sulphur metabolism, metal-handling, secondary metabolism (anthocyanins, dihydroflavonols, carotenoids, non-mevalonate isoprenoids, alkaloids, phenylpropanoids, lignin, GS synthesis and GS degradation), hormones (auxin, brassinosteroids, cytokinin, ethylene, gibberellic acid, and jasmonate), and stress responses (general, biotic, PR proteins, protease inhibitors, defensins, heat shock, touch or wounding, drought and salt, and non-specified abiotic stress) (Additional file 1: Table S1). In addition, more redox-related genes potentially impacting the levels of reactive oxygen species (ROS) were up-regulated in AtGL3+ cotyledons than in K-5-8 cotyledons relative to Westar, although up-regulated redox-related genes unique to K-5-8 cotyledons had very strong transcriptional responses. In addition, genes involved in aspects of development, lipid metabolism, amino acid metabolism, photosynthesis, heat shock, and cold-response were more frequently and more strongly up-regulated in K-5-8 cotyledons (Additional file 1: Table S1).

**Table 2:** Strongly up-regulated stress-responsive genes changes common to transgenic hairy *B. napus* cotyledons

		Expression relative to Westar	
ID	Description	AtGL3 line	K-5-8 line
Biotic Stress			
bo5g027670	CORI1, ATHCOR1, ATCLH1 (CORONATINE-INDUCED 1); chlorophyllase	5.30E+07	21.57
bra008224	pathogenesis-related thaumatin family protein	2.83	14.46
bo2g069600	MLP28 (MLP-LIKE PROTEIN 28)	174.64	13.00
bra007947	"	119.90	11.52
bra016785	RPS5 (RESISTANT TO P. SYRINGAE 5); nucleotide binding	7.66	8.11
bra008667	glycosyl hydrolase family 81 protein	6.06	7.46
bo5g149860	CHAT (acetyl CoA:(Z)-3-hexen-1-ol acetyltransferase)	46.71	5.28
bra011734	ATRCCR | ACD2 (ACCELERATED CELL DEATH 2); red chlorophyll catabolite reductase	4.47	4.47
bo9g163710	glycosyl hydrolase family 81 protein	7.85	3.96
bo07027s010	HRT, RCY1, RPP8 (RECOGNITION OF PERONOSPORA PARASITICA 8); binds nucleotides	3.46	3.84
bra009184	NHL3	3.83	3.57
bo7g087120	avirulence induced gene (AIG) protein, putative	3.49	3.26
Biotic Stress Signalling			
bra031065	TIFY10A | JAZ1 (JASMONATE-ZIM-DOMAIN PROTEIN 1); protein binding	5.35E+14	2.49E+08
bo8g102890	"	369.12	66.76
bo5g027170	"	4.17	12.21
Biotic Stress Regulation of Transcription			
bra027377	RSH2 (RELA-SPOT HOMOLOG 2); GTP diphosphokinase	4.97	5.09
bo5g131760	"	3.24	3.25
PR proteins General			
bo8g091760	disease resistance protein (TIR-NBS-LRR class), putative	5.81	6.67
bra005378	disease resistance family protein	3.09	6.40
bo6g007620	molecular_function unknown; LOCATED IN: endomembrane system	3.94	5.00
bo1g048080	disease resistance protein (NBS-LRR class), putative	1.23	3.75
PR proteins Protease Inhibitors			
bo6g010170	trypsin and protease inhibitor family protein / Kunitz family protein	165.56	3.67
bo6g010250	"	11.99	3.54
bo6g010100	"	113.13	3.33
bra015999	"	78.81	3.25
bra037702	trypsin inhibitor, putative	18.46	1.40
bra016073	trypsin and protease inhibitor family protein / Kunitz family protein	49.47	1.07
Wounding			
bra034157	WI12, SAG20 (SENESCENCE ASSOCIATED GENE 20)	1.07E+301	1.07E+301
bra029887	"	2.93E+05	4.53E+04
bra010381	wound-responsive protein-related	6697.92	76.02
bo7g111980	"	5.51	10.78
bo8g054160	"	6.39	9.56
Abiotic Stress General			
bo01463s030	benzodiazepine receptor-related	1.90E+04	4.85E+05
bra021442	"	798.78	32627.32
bra007841	unknown protein	388.29	266.92
bo8g099690	"	96.36	115.05
bo4g154720	"	2.96	6.57
Heat responsive			
bo1g138440	DNAJ heat shock N-terminal domain-containing protein	1.25E+15	7.51E+23
bo9g026330	DNAJ heat shock protein, putative	6642.31	1.68E+11
bra037247	"	7768.93	1.62E+10
bo7g117750	DNAJ heat shock N-terminal domain-containing protein (J11)	1.46E+08	7.57E+09
bra039384	DNAJ heat shock N-terminal domain-containing protein	1238.27	1.74E+06
bra011656	DNAJ heat shock N-terminal domain-containing protein (J11)	216.64	2.14E+04
bo1g005990	"	415.52	1.04E+04
bra034691	DNAJ heat shock N-terminal domain-containing protein	12.28	139.25
bra017744	DNAJ heat shock N-terminal domain-containing protein (J11)	18.63	77.88
bra020505	DNAJ heat shock protein, putative	8.76E+04	24.75
bo5g132640	DNAJ heat shock N-terminal domain-containing protein	5.58	23.31
bra011735	ATHSF4 | HSF4 (HEAT SHOCK FACTOR 4); DNA binding /transcription repressor	7.14E+04	18.15
bra018216	17.6 kDa class I small heat shock protein (HSP17.6C-CI) (AA 1-156)	47.14	12.92
bo2g158600	DNAJ heat shock protein, putative	788.57	7.39
bo8g066630	Hsp70b (heat shock protein 70B); ATP binding	6.69	7.32
bo4g169420	17.6 kDa class I small heat shock protein (HSP17.6B-CI)	13.24	7.18
bo8g097710	DNAJ heat shock N-terminal domain-containing protein	3.07	5.16
bra016644	Hsp70b (heat shock protein 70B); ATP binding	4.04	4.31
bo1g134560	DNAJ heat shock N-terminal domain-containing protein	8.46	3.95
bo2g029130	heat shock protein-related	4.61	3.81
bra004457	DNAJ heat shock N-terminal domain-containing protein	2.72	3.75
bra003592	J8; heat shock protein binding / unfolded protein binding	4.72	3.70
bra020419	heat shock protein-related	4.29	3.60
bo9g176930	DNAJ heat shock N-terminal domain-containing protein	4.42	3.46
bo3g091720	HSC70-1 (HEAT SHOCK COGNATE PROTEIN 70-1); ATP binding	4.25	3.29
bo8g102330	DNAJ heat shock family protein	4.19	3.10
Cold responsive			
bra017742	CSDP1 (cold shock domain protein 1); RNA/single/double-stranded DNA binding	1160.12	131.40
bo7g117730	"	1.31E+14	8.31
bra013087	WCOR413-LIKE, FL3-5A3 | COR413-PM1	14.61	2.84
Drought and salt responsive			
bo3g052160	early-responsive to dehydration protein-related / ERD protein-related	177.05	1.43E+11
bra039623	ATCOAD (4-phosphopantetheine adenylyltransferase)	4.62	5.42
bo6g004950	QUA2 | TSD2 (TUMOROUS SHOOT DEVELOPMENT 2); methyltransferase	3.50	3.64
bo4g154160	hydrophobic protein, putative / low temperature-salt responsive	3.15	3.24
Abiotic Stress unspecified			
bo8g052730	PHOS34 | universal stress protein (USP) family protein	9.63	35.62
bo2g069600	MLP28 (MLP-LIKE PROTEIN 28)	174.64	13.00
bo7g111350	PHOS34 | universal stress protein (USP) family protein	6.84	12.10
bra007947	MLP28 (MLP-LIKE PROTEIN 28)	119.90	11.52
bra008745	universal stress protein (USP) family protein	4.45	4.83
bo5g002660	ozone-responsive stress-related protein, putative	4.21	4.15
bra022721	PHOS34 | universal stress protein (USP) family protein	4.15	3.76
bo9g165720	"	3.08	3.60
bo3g039740	"	3.31	3.05
bo6g020120	"	3.27	2.94

^a^Cotyledon values are arranged from highest expression to lowest expression within each functional category using the K-5-8 line.

**Table 3:** Trichome-related glabrous cotyledon genes in the hairy leaf AtGL3+ and ultra-hairy leaf K-5-8 lines relative to glabrous leaf *B. napus* cv. Westar

ID	Bin Name	Description	Expression relative to Westar	
			AtGL3 line	K-5-8 line
ESTs common to both transgenic lines				
bo5g002440	Positive initiation	AN (ANGUSTIFOLIA); protein binding	5.39	1.63
bra024875	"	RGA1 (REPRESSOR OF GA1-3 1); protein binding / transcription factor	3.29	1.59
bo9g070200	"	RGA1 (REPRESSOR OF GA1-3 1); protein binding / transcription factor	1.49	0.73
bra007766	"	FDH, KCS10 (3-KETOACYL-COA SYNTHASE 10); acyltransferase	1.51	0.42
bo1g116200	Positive branching	DER1, LSR2, ENL2 | ACT2 (ACTIN 2); structural constituent of cytoskeleton	1.53	1.46
bra020572	"	TUA6; structural constituent of cytoskeleton	1.54	0.73
bra039648	"	TUA6; structural constituent of cytoskeleton	1.48	0.55
bo1g054880	"	SPK1 (SPIKE1); GTP binding / GTPase binding / guanyl-nucleotide exchange factor	1.51	0.47
bra009451	Multicellular trichomes	SIM (SIAMESE); cyclin-dependent protein kinase inhibitor	1978.24	11.69
bo9g178800	"	SIM (SIAMESE); cyclin-dependent protein kinase inhibitor	935.76	7.64
bra027928	Less developed	unknown protein	1.45	0.42
bo1g046580	"	MRH5, GPDL2 | SHV3 (SHAVEN 3); glycerophosphodiester phosphodiesterase/ kinase	1.14	0.37
bra026409	"	MRH5, GPDL2 | SHV3 (SHAVEN 3); glycerophosphodiester phosphodiesterase/ kinase	1.13	0.35
bo9g054590	"	unknown protein	1.44	0.24
ESTs unique to the AtGL3+ line				
bra033258	Positive initiation	AN (ANGUSTIFOLIA); protein binding	14.72	NA
bra017443	"	RGA1 (REPRESSOR OF GA1-3 1); protein binding / transcription factor	1.47	NA
bra029388	Distorted	EMB3009 (embryo defective 3009); transferase/ transferase, transferring acyl groups	1.07E+301	NA
bo7g097360	"	EMB3009 (embryo defective 3009); transferase/ transferase, transferring acyl groups	1.05E+59	NA
bra004054	Positive branching	TPS6 | ATTPS6; alpha,alpha-trehalose-phosphate synthase (UDP-forming)/ transferase	3.29	NA
bo3g071760	"	DER1, LSR2, ENL2 | ACT2 (ACTIN 2); structural constituent of cytoskeleton	1.68	NA
bra022356	"	DER1, LSR2, ENL2 | ACT2 (ACTIN 2); structural constituent of cytoskeleton	1.64	NA
bo5g117040	"	DER1, LSR2, ENL2 | ACT2 (ACTIN 2); structural constituent of cytoskeleton	1.55	NA
bra037560	"	DER1, LSR2, ENL2 | ACT2 (ACTIN 2); structural constituent of cytoskeleton	1.48	NA
bra008705	Negative branching	ATMIXTA | ATMYB16 (MYB DOMAIN PROTEIN 16); DNA binding / transcription factor	5.94	NA
bo5g021100	Endoreduplication	CYCA2;3 (CYCLIN A2;3); cyclin-dependent protein kinase regulator	1.39	NA
bo5g038710	Trichome size	ATSAC1 (suppressor of actin 1); phosphatidylinositol-4,5-bisphosphate 5-phosphatase	7.73	NA
bo6g027740	Less developed	MRH5, GPDL2 | SHV3 (SHAVEN 3); glycerophosphodiester phosphodiesterase/ kinase	38.59	NA
ESTs unique to the K-5-8 line				
bo8g115250	Positive initiation	HDG2 (HOMEODOMAIN GLABROUS 2); DNA binding / transcription factor	NA	0.43
bo8g067530	"	HDG12 (HOMEODOMAIN GLABROUS 12); transcription factor	NA	0.27
bra015401	"	HDG2 (HOMEODOMAIN GLABROUS 2); DNA binding / transcription factor	NA	0.13
bo6g029470	Positive branching	TPS6 | ATTPS6; alpha,alpha-trehalose-phosphate synthase (UDP-forming)/ transferase	NA	1.65
bo6g107020	"	TUA6; structural constituent of cytoskeleton	NA	0.63
bo9g022270	"	PKCBP, KCBP | ZWI (ZWICHEL); calmodulin binding / microtubule motor	NA	0.57
bra018825	"	TUA6; structural constituent of cytoskeleton	NA	0.53
bra016164	Negative initiation	MYBL2 (ARABIDOPSIS MYB-LIKE 2); DNA binding / transcription factor	NA	1.56
bo3g149420	"	UPL3 | KAK (KAKTUS); ubiquitin-protein ligase | chr4:18041031-18049292 REVERSE'	NA	0.68
bo2g012520	Negative branching	ATMIXTA | ATMYB16 (MYB DOMAIN PROTEIN 16); DNA binding / transcription factor	NA	0.36
bo3g010230	"	ATMIXTA | ATMYB16 (MYB DOMAIN PROTEIN 16); DNA binding / transcription factor	NA	0.29
bo9g164230	"	ATMIXTA | ATMYB16 (MYB DOMAIN PROTEIN 16); DNA binding / transcription factor	NA	0.18
bra024337	Trichome size	HYS1 | CPR5 (CONSTITUTIVE EXPRESSION OF PR GENES 5)	NA	1.94
bo2g029050	"	FLP1, YRE, CER3, WAX2 | CER3 (ECERIFERUM 3); binding / catalytic/ iron ion binding /oxidoreductase	NA	0.56
bra020412	"	FLP1, YRE, CER3, WAX2 | CER3 (ECERIFERUM 3); binding / catalytic/ iron ion binding /oxidoreductase	NA	0.41
bo9g133540	"	FLP1, YRE, CER3, WAX2 | CER3 (ECERIFERUM 3); binding / catalytic/ iron ion binding /oxidoreductase	NA	0.20

^a^Cotyledon values are arranged from highest expression to lowest expression within each functional category using the K-5-8 line

### Expression of trichome genes in glabrous cotyledons of hairy-leaf and ultrahairy-leaf lines

The transgenic cotyledons remained glabrous despite the expression of *AtGL3* or in conjunction with the manipulation of *BnTTG1* expression (2). Hence, transcript levels for all known trichome genes were compared in both types of transgenic cotyledons to those of cv. Westar. According to RNA sequencing, no trichome genes were down-regulated in the glabrous transgenic cotyledons relative to cv. Westar cotyledons, and up-regulation of most transcripts involved trichome genes that should impact trichome structure (Table 3). Overall, the greatest change in transcript level relative to cv. Westar cotyledons included two *SIAMESE* (*SIM*) genes (bra009451 and bo9g178890) specifying multi-cellular trichomes that were also more highly expressed in the AtGL3+ line than in the K-5-8 line (Table 3). As well, two *embryo defective 3009* genes (EMB3009) encoding acyl transferases involved in trichome shape were highly up-regulated in the AtGL3+ line, while *SAC1* (affecting trichome size), *SHAVEN3*, and the *MYB16 MIXTA* genes were also up-regulated in this line (Table 3). A paralogue of the *BrRGA1* gene was uniquely up-regulated in the AtGL3+ cotyledons and two *RGA1* genes were commonly up-regulated in both transgenic lines.

**Fig. 5:**
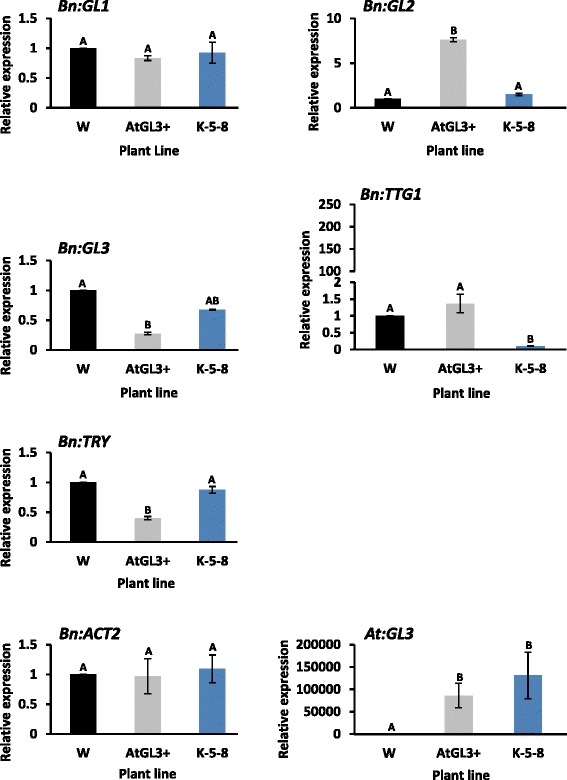
qRT-PCR analysis of expression levels of five trichome regulatory genes encoding elements of the MBW tri-protein complex in glabrous cotyledons of 10-day-old hairy leaf (AtGL3+) and ultra-hairy leaf (K-5-8) *B. napus* lines relative to cv. Westar (W). Level of *BnACT2* and *AtGL3* are provided as controls. Plants were grown under a 16/8 h diurnal light cycle

Well-known trichome genes coding for the MBW tri-protein initiation complex were not differentially expressed in 7-day-old cotyledons. Only one well-known trichome regulatory gene, *ANGUSTIFOLIA* (AN, affecting trichome initiation), had a significantly different transcript profile in AtGL3+ or K-5-8 cotyledons compared with cv. Westar (Table 3). qRT-PCR with 10-day-old cotyledons showed that five well-known MBW trichome genes were weakly expressed. Although the *BnGL2* trichome-initiation gene was expressed at a higher level in AtGL3+ cotyledons compared with K-5-8 and Westar cotyledons, *BnGL3* and *BnTRY* were expressed at lower levels, while changes for *BnTTG1* (which was manipulated) occurred only in the K-5-8 line (Fig. 5) as expected.

## Discussion

This study examined the composition of glabrous cotyledons from a hairy leaf line derived from the introduction of the *AtGL3* gene (line AtGL3+) into *B. napus* [1] and an ultra-hairy leaf line derived by repression of *BnTTG1* within the AtGL3+ *B. napus* background (line K-5-8) [2]. Expression of *AtGL3* induces the formation of trichomes on young leaves of these lines [1, 2] which reduces the insect’s ability to physically interact with the host plant and reduces FB feeding damage; however, the glabrous cotyledons of these lines also deter FB feeding [21]. A comparison of the physiological properties and composition of the cotyledons (Table 4) provides a few clues as to whether a common mechanism(s) might be responsible for the antixenosis. In summary, the cotyledons of the AtGL3+ have an abnormal appearance, which is in keeping with vegetative tissue in this line, but develop normally in the K-5-8 line due to repression of *BnTTG1* expression (2). The cotyledons in the wild-type line are horizontally-oriented; however, the cotyledons in both transgenic lines are vertically-oriented and it was suggested that this orientation may be less suitable for FBs to initiate feeding behaviour [19]. Anthocyanin content in the AtGL3+ line was significantly increased, but reduced in the K-5-8 line compared to wild-type. Both lines had similar cell wall carbohydrate contents; however, cell wall lignin was reduced in the K-5-8 line. While total GS content was reduced in the AtGL3+ line, the level of gluconapin was increased and 4-methoxy-glucobrassicin was decreased in both transgenic lines.

**Table 4:** Summary of physiological properties and composition of *B. napus* cotyledons expressing AtGL3

A. Insect behaviour and cotyledon biochemistry relative to Westar							
Genotype	FB Feeding	Cotyledon Morphology	Cotyledon Orientation	Anthocyanin Accumulation	Cell Wall Carbohydrates	Cell Wall Lignin	
AtGL3+^a^	Decreased	Abnormal	Vertical	Increased	No Difference	No Difference	
K-5-8^b^	Decreased	Normal	Vertical	Decreased	No Difference	Decreased	
B. Glucosinolate composition of transgenic lines relative to Westar							
Genotype	Total GS	Progoitrin^c^	Glucoraphanin^c^	Gluconapin^c^	4-hydroxy-glucobrassicin^c^	Glucobrassicin^c^	4-methoxy-glucobrassicin^c^
AtGL3+	Decreased	No Diference	No Difference	Increased	No Difference	No Difference	Decreased
K-5-8	No Difference	No Difference	No Difference	Increased	Decreased	No Difference	Decreased
C. Glucosinolate composition of the transgenic lines relative to one another.							
Genotype	Total GS	Progoitrin^c^	Glucoraphanin^c^	Gluconapin^c^	4-hydroxy-glucobrassicin^c^	Glucobrassicin^c^	4-methoxy-glucobrassicin^c^
AtGL3+	Decreased	Decreased	No Difference	Increased	No Difference	Increased	Decreased
K-5-8	-	-	-	-	-	-	-

^a^AtGL3+, *B. napus* cv. Westar expressing *AtGL3* under direction of CaMV *35S* promoter

^b^K-5-8, *B. napus* cv. Westar expressing *AtGL3* under direction of CaMV *35S* promoter as well as a *TTG1* RNAi construct

^c^Progoitrin, 2-hydroxy-3-butenyl-GS; Glucoraphanin, 4-methylsulfinylbutyl-GS; Gluconapin, 3-butenyl-GS; 4-hydroxyglucobrassicin, 4-hydroxy-3-indolylmethyl-GS; Glucobrassicin, 3-indolylmethyl-GS; 4-methoxyglucobrassicin, 4-methoxy-3-indolylmethyl-GS

### Role of secondary metabolites in cotyledon resistance

Feeding is highly influenced by GS content for many crucifer specialists; however, there is little consensus as to which GSs stimulate and which deter feeding. Related to the GSs found in cotyledons in the current study, decreased quantities of glucoraphanin and increased levels of progoitrin correlated with increased FB feeding in *Sinapis alba* [31]. In a broad study of various *Brassica* species, progoitrin levels were also correlated with stimulation of FB feeding [32]; however, this does not explain the reduced FB feeding on the transgenic cotyledons in this study as progoitrin and glucoraphanin levels were similar to the wild-type line. The same study and studies with the stem FB (*Psylliodes chrysocephala*) [33, 34] reported that gluconapin is also a feeding stimulant; however, this GS was elevated in cotyledons of both transgenic lines in the current study. Interestingly, glucobrassicin was the most stimulatory of the GS tested in the study with *P. chrysocephala* [33], but levels of this GS were the same in the transgenic and wild-type lines. To our knowledge, no studies have implicated 4-methoxy-glucobrassicin in affecting FB feeding activity. Future experiments appear to warrant examining individual GS to determine, for example, if applying gluconapin further increases resistance or if adding 4-methoxy-glucobrassicin restores wild-type predation by FB in the transgenic cotyledons.

It has been difficult to extrapolate the response to individual GSs in laboratory studies to damage occurring in the field [35, 36] as this is influenced by the presence of other GSs and other plant secondary metabolites, as well as the myriad GS-derived volatiles that result from the release of myrosinase and associated myrosinase-specifier proteins upon tissue disruption [37]. Communication and recruitment of con-specific insects must also be considered when assessing host susceptibility. In the field, allyl isothiocyanate derived from the GS sinagrin is a strong FB attractant [38]. This volatile also enhances the response of FBs to aggregation pheromone [39], which functions only in the presence of specific host plant volatiles [40]. A role for flavonoids in host recognition and acceptance by many adult insects has also been reported [41, 42]. Moreover, anthocyanins are known to impact insect feeding [28]. Since anthocyanin production was altered in the AtGL3+ cotyledons, these should also be tested for their impact on FB feeding.

### Impact of *AtGL3* expression on the cotyledon transcriptome

AtGL3+ cotyledon size and colour (small with a dark red abaxial side) differ from that of K-5-8 or Westar, and AtGL3+ plants are smaller and grow less vigorously [2, 18]. Not surprisingly, the alteration of the cotyledon transcriptome in the AtGL3+ plants was more extreme (both in transcript diversity and expression intensity) compared with K-5-8 cotyledons (both lines relative to the Westar cotyledon transcriptome). In contrast, changes to the K-5-8 cotyledon transcriptome were less extreme (relative to AtGL3+ cotyledons), which may reflect the healthier cotyledons that were similar in size to Westar cotyledons and exhibited more vigorous growth [2, 18]. The cell wall of K-5-8 cotyledons was also less lignified suggesting that K-5-8 cotyledons could be in a more vigorous growth phase than the AtGL3+ cotyledons. The red anthocyanin present in AtGL3+ cotyledons growing under continuous light appeared to be part of a stronger and more varied stress response as indicated by the transcriptome data. In addition to the up-regulation of genes directly involved in anthocyanin production, genes involved in hormone synthesis or signalling (jasmonate, auxins, gibberellins, brassinosteroids, and ethylene) were also more strongly up-regulated in the AtGL3+ cotyledons than in those from K-5-8, while genes involved in development, lipid synthesis, amino acid metabolism, and photosynthesis were more strongly up-regulated in K-5-8. These data suggest that AtGL3+ cotyledons are compromised and less able to fuel growth, while K-5-8 cotyledons are primed for better growth characteristics and possibly more able to tolerate FB damage.

### Expression of genes involved in trichome formation

Cotyledons of lines expressing *AtGL3* remained glabrous; however, RNA sequencing showed that both types of transgenic cotyledons had elevated levels of transcripts for many genes involved in trichome synthesis. *SIM*, which encodes a cyclin-dependent protein kinase inhibitor specifying multi-cellular trichomes, was up-regulated in these cotyledons. As well, the AtGL3+ line had an elevated level of transcripts for *EMB3009* which specifies trichome shape, *AUGUSTIFOLIA* which is involved in trichome initiation, *MIXTA* which specifies negative trichome branching, *SAC1* which specifies trichome size, and SHAVEN3 which specifies trichome number. These differences may underlie the smaller trichomes present on the AtGL3+ line [2]. Moreover, a paralogue of the *BrRGA1* gene was uniquely up-regulated in the AtGL3+ cotyledons, and other *RGA1* genes were up-regulated in cotyledons of both transgenic lines. RGA1 (repressor of gibberellic acid 1) is a GA-insensitive DELLA repressor protein that negatively impacts the activity of trichome transcriptional activators [43]. Hence, induction of this repressor could be one of the reasons why the transgenic cotyledons remained glabrous even in the presence of a high *AtGL3* transcript levels. Induction of *RGA1* also suggests that it could be a cotyledon-specific gene, since these repressors do not appear in the leaf transcriptome of either transgenic line, or in leaves of cv. Westar [2]. The data implies that the expression of the *AtGL3* gene in the two transgenic lines may have a measure of positive control by stimulating over-expression of this trichome repressor, ensuring that cotyledons will be maintained as sources of nutrients for young seedlings rather than becoming trichome-bearing leaf-like organs. This is supported by the fact that five trichome regulatory genes related to the MBW initiation complex were only weakly expressed in cotyledons.

Shared components within the MBW regulatory complex link trichome formation and anthocyanin biosynthesis. In trichome formation, GL3 or ENHANCER OF GL3 forms a complex with TTG1 and the MYB protein GL1. This complex activates the expression of genes encoding transcription factors that in turn induce trichome formation. Anthocyanin biosynthesis is regulated by a complex consisting of TTG1 and GL3, but with different MYB factors [17]. In the current study, the chemical profile and changes in the expression of genes in cotyledons expressing *AtGL3* alone (AtGL3+) or accompanied by reduced *BnTTG1* expression (K-5-8) suggest that the production of other secondary metabolites, such as GSs, may be subject to a similar type of regulation. At least 6 other MYB proteins (CPC, TRY, ETC1, ETC2, ETC3 and TCL1) can replace GL1 and alter the specificity of the regulatory complex [15]. In the future, it would interesting to establish if some type of MBW complex also regulates GS production and what MYB protein(s) might be involved.

## Conclusion

An unexpected outcome of manipulating the expression of *AtGL3* and *BnTTG1* genes was cotyledon resistance to FB, in spite of the fact that this tissue remained glabrous [18, 19]. As such, these glabrous cotyledon transcriptomes represent roadmaps that can lead to the identification of insect-resistance compounds and properties. The altered host interactions could reflect the large up-regulation of genes affecting the synthesis of GSs, other secondary metabolites and tissue structural components, including cell wall carbohydrates, lignin, wax, metal handling systems, flavonoids, phenolics, and indole alkaloids. Quercetin (flavonoid) and chlorogenic acid (phenolic) derivatives accumulate strongly in cotyledons of the crucifer *Camelina sativa*, protecting them from FB feeding [41], while *B. napus* cotyledons normally accumulate only traces of these compounds (Gruber unpublished). Seeds of the crucifer *Lunnaria annua* L. (*L. bioennis*) accumulate toxic lunarine/lunaridin alkaloids [44], and alkaloids have been shown to deter insects. Plants that accumulate certain metals in the Brassicaceae can also be more resistant to specific insects [45–51]. Finally, the induction of large numbers stress-responsive genes (specifying wounding, abiotic, and biotic stress responses), such as those encoding heat shock proteins, PR proteins, protease inhibitors, glucosinolate synthesis/breakdown factors, abiotic stress factors, redox proteins, transcription factors, and proteins required for auxin metabolism suggest that these cotyledons may now be primed for resistance to other forms of biotic and abiotic stress.

## Additional files

Additional file 1 Table S1. MAPMAN functional categories of up-regulated genes. Organization of up-regulated genes from AtGL3+ and K-5-8 cotyledons compared to *B. napus* cv. Westar into 36 functional categories (BINS) according to MAPMAN [24]. (XLS 3406 kb)

Additional file 2 Table S2. Genes specifying glucosinolates and their degradation products. Up-regulated genes from AtGL3+ and K-5-8 cotyledons compared to *B. napus* cv. Westar involved in aspects of glucosinolate biosynthesis or degradation. (DOCX 27 kb)

Additional file 3 Figure S1. MAPMAN (heat map) functional overview of changes in gene expression in K-5-8 glabrous cotyledons. MAPMAN (heat map) functional overview of changes in gene expression in glabrous cotyledons in the 10-day-old hairy leaf (K-5-8) *B. napus* line relative to cv. Westar. The 36 BINs represent MAPMAN sub-cellular function categories. [7497 out of 8037 differentially expressed genes were mapped using this method, with a few genes mapped into more than one category.] The majority of changes involved up-regulated genes. Blue blocks represent individual up-regulated genes. Red blocks represent 29 individual down-regulated genes. The full spectrum of Category 35 genes (unknowns) was too large to fit on the figure. Relative expression intensity scale is in log2, where darkest colour intensity represents log_2_5 and higher/(+ 5) or lower (− 5) relative to Westar. (PPT 484 kb)

Additional file 4 Figure S2. MAPMAN (heat map) functional overview of changes in gene expression in AtGL3+ glabrous cotyledons. MAPMAN (heat map) functional overview of changes in gene expression in glabrous cotyledons in the 10-day-old hairy leaf (AtGL3+) *B. napus* line relative to cv. Westar. The 36 BINs represent MAPMAN sub-cellular function categories. [8186 out of 8841 differentially expressed genes were mapped using this method, with a few genes mapped into more than one category.] The majority of changes involved up-regulated genes. Blue blocks represent individual up-regulated genes. Red blocks represent 29 individual down-regulated genes. The full spectrum of Category 35 genes (unknowns) was too large to fit on the figure. Relative expression intensity scale is in log2, where darkest colour intensity represents log_2_5 and higher/(+ 5) or lower (− 5) relative to Westar. (PPT 293 kb)

Additional file 5 Figure S3. MAPMAN heat maps of stress responsive genes in glabrous cotyledons. MAPMAN heat maps of stress responsive genes in glabrous cotyledons of (A) 10-day-old hairy leaf (AtGL3+) and (B) ultra hairy leaf (K-5-8) *B. napus* lines relative to cv. Westar. Blue and red blocks represent individual up- and down-regulated genes. Relative expression intensity scale is in log_2_ where ±4 represents ± log_2_4 or greater. (PPT 401 kb)

Additional file 6 Figure S4. MAPMAN heat maps of metabolism genes in glabrous cotyledons. MAPMAN heat maps of metabolism genes in glabrous cotyledons of (A) hairy leaf AtGL3+ *B. napus* and (B) ultra-hairy leaf K-5-8 *B. napus,* relative to Westar. Maps show numbers of ESTs and expression intensity. Blue blocks represent up-regulated genes. Red blocks represent individual down-regulated genes. Relative expression intensity scale is in log_2_, where ±5 represents ±log_2_5 or greater. (PPT 287 kb)

Additional file 7 Figure S5. MAPMAN heat maps of gene regulation and protein-related genes in glabrous cotyledons. MAPMAN heat maps of gene regulation and protein-related genes in glabrous cotyledons of (A) AtGL3+ *B. napus* and (B) K-5-8 relative to Westar. Maps show numbers of changed ESTs and expression intensity. Blue blocks represent individual up-regulated genes. Red blocks represent individual down-regulated genes. Relative expression intensity scale is in log2 where ±5 represents ±log_2_4 or greater. (PPT 279 kb)
