# Supplementary material for: The biochemical composition and transcriptome of cotyledons from Brassica napus lines expressing the AtGL3 transcription factor and exhibiting reduced flea beetle feeding
Source: BMC Plant Biol. 2018 Apr 16;18:64. doi: 10.1186/s12870-018-1277-6 (PMC5902958; doi:10.1186/s12870-018-1277-6)
Supplement: Supplementary file 2 — Table S2. Genes specifying glucosinolates and their degradation products. Up-regulated genes from AtGL3+ and K-5-8 cotyledons compared to B. napus cv. Westar involved in aspects of glucosinolate biosynthesis or degradation. (DOCX 27 kb) [file 12870_2018_1277_MOESM2_ESM.docx]

| **Table S2: Genes specifying glucoinolates and their degradation products** | | | | |
| --- | --- | --- | --- | --- |
| ***B. napus* Genome ID** | | **Description of Arabidopsis homologue and AT chomosome location** | **^1^Fold Expression relative to Westar** | |
| **ESTs common to the AtGL3+ line and the K-5-8 line** | | | **AtGL3+** | **K-5-8** |
| ***Biosynthesis*** | | |  |  |
| bra027623 | | ATST5B \| SOT18 (DESULFO-GLUCOSINOLATE SULFOTRANSFERASE 18); aliphatic 3/4/5/7-methylthiopropyl/butyl/pentyl/heptyl-desulfoglucosinolate sulfotransferase \| chr1:27862909-27864193 FORWARD | 6.16E+04 | 3.53E+05 |
| bo9g028880 | | " | 2179.83 | 354.59 |
| bra036703 | | HLS3, ALF1, RTY1 \| SUR1 (SUPERROOT 1); S-alkylthiohydroximate (C-S) lyase/transaminase \| chr2:8877960-8880388 REVERSE | 3.41 | 8.22 |
| bo2g080910 | | CORI-7, ATST5A \| SOT16 (SULFOTRANSFERASE 16); indole desulfoglucosinolate sulfotransferase \| chr1:27864345-27865694 REVERSE | 37.01 | 4.17 |
| bra008132 | | " | 18.38 | 3.32 |
| bra027035 | | FMO GS-OX2 (FLAVIN-MONOOXYGENASE GLUCOSINOLATE S-OXYGENASE 2); aliphatic 3/4/5/7/8-methylthiopropyl glucosinolate S-oxygenase \| chr1:23151794-23155717 FORWARD | 1.27 | 1.45 |
| bo9g037180 | | " | 1.34 | 1.30 |
| bo9g050970 | | ATST5B \| SOT18 (DESULFO-GLUCOSINOLATE SULFOTRANSFERASE 18); aliphatic 3/4/5/7-methylthiopropyl/butyl/pentyl/heptyl-desulfoglucosinolate sulfotransferase \| chr1:27862909-27864193 FORWARD | 1.12 | 1.27 |
| bo7g118840 | | CYP79B2; electron carrier/ heme- iron ion-oxygen binding / indole monooxygenase \| chr4:18525246-18527579 FORWARD | 4.86 | 1.17 |
| bo2g161590 | | HAG1 \| MYB28 (myb domain protein 28); DNA binding / aliphatic transcription factor \| chr5:24689151-24690797 REVERSE | 1.35 | 1.15 |
| bra029311 | | " | 1.41 | 1.15 |
| bo4g049480 | | UDP-glucoronosyl/aliphatic UDP-glucosyl transferase family protein \| chr2:13518211-13520364 FORWARD | 1.37 | 1.06 |
| bo1g002970 | | CYP79B2; electron carrier/ heme- iron ion-oxygen binding / indole monooxygenase \| chr4:18525246-18527579 FORWARD | 20.11 | 1.06 |
| ***Degradation*** | | |  |  |
| bo7g089110 | | NSP5 (NITRILE SPECIFIER PROTEIN 5) \| chr5:19540937-19542426 REVERSE | 13.45 | 4.01E+04 |
| bra037506 | | " | 6.02 | 99.04 |
| bo6g093940 | | myrosinase-associated protein, putative \| chr1:20161670-20163743 REVERSE | 2.49E+03 | 4.38 |
| bra037954 | | " | 77.71 | 2.91 |
| bo7g067500 | | TASTY \| ESP (EPITHIOSPECIFIER PROTEIN); enzyme regulator \| chr1:20170715-20173949 REVERSE | 113.77 | 2.22 |
| bra035006 | | NIT2 (nitrilase 2); indole-3-acetonitrile nitrile hydratase chr3:15983311-15985535 FORWARD | 1.64 | 1.56 |
| bo7g067530 | | TASTY \| ESP (EPITHIOSPECIFIER PROTEIN); enzyme regulator \| as above | 15.56 | 1.55 |
| bo00934s010 | | BGLU38 \| TGG1 myrosinase (THIOGLUCOSIDE GLUCOHYDROLASE 1) chr5:9079505-9082384 REVERSE | 33.82 | 1.48 |
| bra030404 | | MBP1.2, F-ATMBP \| MBP2 (MYROSINASE-BINDING PROTEIN 2); sugar/thioglucosidase binding \| chr1:19345940-19348668 REVERSE | 1.36 | 1.47 |
| bra039823 | | BGLU38 \| TGG1 myrosinase (THIOGLUCOSIDE GLUCOHYDROLASE 1) \| as above | 80.45 | 1.30 |
| bra039824 | | " | 80.45 | 1.30 |
| bo2g155840 | | " | 11.08 | 1.30 |
| bra032343 | | " | 11.24 | 1.29 |
| bra020523 | | " | 35.51 | 1.29 |
| bo6g093870 | | TASTY \| ESP (EPITHIOSPECIFIER PROTEIN); enzyme regulator \| as above | 205.07 | 1.28 |
| bo2g155870 | | BGLU38 \| TGG1 myrosinase (THIOGLUCOSIDE GLUCOHYDROLASE 1) \| as above | 22.47 | 1.27 |
| bra020551 | | " | 27.47 | 1.22 |
| bo3g045170 | | BGLU37 \| TGG2 myrosinase (GLUCOSIDE GLUCOHYDROLASE 2) chr5:9072727-9075690 FORWARD | 5.35 | 1.21 |
| bra037958 | | TASTY \| ESP (EPITHIOSPECIFIER PROTEIN); enzyme regulator \| as above | 44.32 | 1.20 |
| bo5g126100 | | Symbols: ATMLP-470, NSP1, ATNSP1 \| NSP1 (NITRILE SPECIFIER PROTEIN 1) \| chr3:5566086-5568545 FORWARD | 1.13 | 1.17 |
| bra022157 | | " | 1.17 | 1.16 |
| bra014309 | | MBP1.2, F-ATMBP \| MBP2 (MYROSINASE-BINDING PROTEIN 2); sugar / thioglucosidase binding \|as above | 1.33 | 1.15 |
| bo14804s010 | | BGLU37 \| TGG2 myrosinase (GLUCOSIDE GLUCOHYDROLASE 2) as above | 6.54 | 1.10 |
| bra027359 | | ESM1 (epithiospecifier modifier 1); carboxylesterase/ hydrolase \| chr3:4729823-4731803 FORWARD | 1.09 | 1.09 |
| bo9g023670 | | BGLU38 \| TGG1 myrosinase (THIOGLUCOSIDE GLUCOHYDROLASE 1) \| as above | 3.68 | 1.08 |
| bo8g063740 | | " | 3.27 | 1.08 |
| bo3g181200 | | NSP4 (NITRILE SPECIFIER PROTEIN 4) \| chr3:5571992-5574541 FORWARD | 1.11 | 1.06 |
| bo5g131590 | | ESM1 (epithiospecifier modifier 1); carboxylesterase/ hydrolase, acting on ester bonds \| as above | 1.09 | 1.06 |
| bo3g181210 | | NSP4 (NITRILE SPECIFIER PROTEIN 4) \| above | 1.26 | 1.03 |
| bra004012 | | BGLU38 \| TGG1 myrosinase (THIOGLUCOSIDE GLUCOHYDROLASE 1) \| as above | 22.63 | 1.02 |
| **ESTs unique to the AtGL3+ line** | | |  |  |
|  | | ***Biosynthesis*** |  |  |
| bo5g025610 | | ATST5C \| SOT17 (SULFOTRANSFERASE 17); aliphatic desulfoglucosinolate sulfotransferase \| chr1:6398580-6399942 FORWARD | 9877.98 | NA |
| bra009100 | | CYP79A2 (CYTOCHROME P450 79A2); aromatic oxidoreductase, acting on paired donors (NADH or NADPH as one donor), incorporation or reduction of molecular oxygen, oxygen binding \| chr5:1559778-1561765 REVERSE | 1758.34 | NA |
| bra010644 | | CYP79B2; electron carrier/ heme-iron ion-oxygen binding / indole monooxygenase \| chr4:18525246-18527579 FORWARD | 76.64 | NA |
| bo6g008450 | | CORI-7, ATST5A \| SOT16 (SULFOTRANSFERASE 16); indole desulfoglucosinolate sulfotransferase \| chr1:27864345-27865694 REVERSE | 48.17 | NA |
| bo3g152800 | | CYP79B2; electron carrier/ heme- iron ion-oxygen binding / indole monooxygenase \| chr4:18525246-18527579 FORWARD | 35.75 | NA |
| bo2g161180 | | ATR1, MYB34 \| MYB34 (MYB DOMAIN PROTEIN 34); kinase/ indole transcription activator \| chr5:24494691-24496351 FORWARD | 17.75 | NA |
| bra024634 | | UGT74B1 (UDP-glucosyl transferase 74B1); shared UDP-glycosyltransferase/ thiohydroximate beta-D-glucosyltransferase \| chr1:8525435-8527087 REVERSE | 14.52 | NA |
| bo9g177260 | | CYP79A2 (CYTOCHROME P450 79A2); aromatic oxidoreductase, acting on paired donors (NADH or NADPH as one donor), incorporation or reduction of molecular oxygen, oxygen binding \| chr5:1559778-1561765 REVERSE | 12.64 | NA |
| bra036490 | | HLS3, ALF1, RTY1 \| SUR1 (SUPERROOT 1); S-alkylthiohydroximate (C-S) lyase/ transaminase \| chr2:8877960-8880388 REVERSE | 10.27 | NA |
| bra011821 | | CYP79B2; electron carrier/ heme-iron ion-oxygen binding / indole monooxygenase \| chr4:18525246-18527579 FORWARD | 10.20 | NA |
| bo5g041080 | | UGT74B1 (UDP-glucosyl transferase 74B1); shared UDP-glycosyltransferase/ thiohydroximate beta-D-glucosyltransferase \| chr1:8525435-8527087 REVERSE | 9.71 | NA |
| bra016908 | | REF2 \| CYP83A1 (CYTOCHROME P450 83A1); aliphatic oxidoreductase, acting on paired donors, NADH or NADPH as one donor, and incorporation of one atom of oxygen / oxygen binding \| chr4:7990485-7992311 REVERSE | 6.15 | NA |
| bo7g003330 | | HLS3, ALF1, RTY1 \| SUR1 (SUPERROOT 1); S-alkylthiohydroximate (C-S) lyase/ transaminase \| chr2:8877960-8880388 REVERSE | 3.81 | NA |
| bo8g078930 | | BCAT3 (BRANCHED-CHAIN AMINOTRANSFERASE 3); aliphatic branched-chain-amino-acid transaminase/ catalytic \| chr3:18422649-18425693 FORWARD | 1.48 | NA |
| bo1g080200 | | " | 1.47 | NA |
| bo8g067910 | | BW51A or B, HIG1 \| MYB51 (MYB DOMAIN PROTEIN 51); DNA binding /indole transcription factor \| chr1:6389411-6391267 FORWARD | 1.29 | NA |
| ***Degradation*** | | |  |  |
| bra000847 | AOP1.1 \| AOP1; oxidoreductase, acting on paired donors (2-oxoglutarate as one donor), incorporating one oxygen atom into both donors \| chr4:1358432-1359698 FORWARD | | 1.07E+301 | NA |
| bo3g181260 | MBP1.2, F-ATMBP \| MBP2 (MYROSINASE-BINDING PROTEIN 2); sugar / thioglucosidase binding \| chr1:19345940-19348668 REVERSE | | 1.07E+301 | NA |
| bra037957 | myrosinase-associated protein, putative \| chr1:20161670-20163743 REVERSE | | 34.78 | NA |
| bo9g022660 | BGLU38 \| TGG1 myrosinase (THIOGLUCOSIDE GLUCOHYDROLASE 1) \| chr5:9079505-9082384 REVERSE | | 26.35 | NA |
| bra039702 | TASTY \| ESP (EPITHIOSPECIFIER PROTEIN); enzyme regulator \| chr1:20170715-20173949 REVERSE | | 24.93 | NA |
| bo6g093890 | myrosinase-associated protein, putative \| chr1:20161670-20163743 REVERSE | | 17.51 | NA |
| bo4g178080 | NSP2 (NITRILE SPECIFIER PROTEIN 2) \| chr2:14029190-14031074 REVERSE | | 6.50 | NA |
| bo4g023800 | BGLU26 \| PEN2 (PENETRATION 2); myrosinase hydrolase, thioglucosidase hydrolyzing O-glycosyl compounds \| chr2:18364756-18367725 FORWARD | | 1.61 | NA |
| **ESTs unique to the K-5-8 line** | | |  |  |
|  | ***Biosynthesis*** | |  |  |
| bo1g105680 | aliphatic aconitase C-terminal domain-containing protein \| chr3:21797058-21798303 REVERSE | | NA | 504.95 |
| bra021743 | aliphatic UDP-glucoronosyl/UDP-glucosyl transferase family protein \| chr2:13518211-13520364 FORWARD | | NA | 2.50 |
| bo2g095850 | aliphatic 3-isopropylmalate dehydrogenase, chloroplast, putative \| chr1:30287763-30290298 FORWARD | | NA | 1.61 |
| bra005641 | aliphatic UDP-glucoronosyl/UDP-glucosyl transferase family protein \| chr2:13518211-13520364 FORWARD | | NA | 1.58 |
| bo8g024390 | SUR2, RNT1, RED1, ATR4 \| CYP83B1 (CYTOCHROME P450 MONOOXYGENASE 83B1); shared oxidoreductase acting on paired donors (NADH or NADPH as one donor), incorporation or reduction of molecular oxygen /oxygen binding \| chr4:15273471-15275310 REVERSE | | NA | 1.38 |
| bra035169 | aliphatic 3-isopropylmalate dehydrogenase, chloroplast, putative \| chr1:30287763-30290298 FORWARD' | | NA | 1.37 |
| bra034941 | " | | NA | 1.28 |
| bo9g175680 | Symbols: MYB29, ATMYB29, PMG2 \| ATMYB29 (ARABIDOPSIS THALIANA MYB DOMAIN PROTEIN 29); DNA binding / shared transcription factor \| chr5:2446764-2448543 FORWARD | | NA | 1.12 |
| bra032734 | REF2 \| CYP83A1 (CYTOCHROME P450 83A1); aliphatic oxidoreductase, acting on paired donors, NADH or NADPH as one donor, and incorporation of one atom of oxygen / oxygen binding \| chr4:7990485-7992311 REVERSE | | NA | 1.09 |
| bo4g130780 | " | | NA | 1.07 |
| bra004744 | aliphatic aconitase C-terminal domain-containing protein \| chr2:17920660-17921689 FORWARD | | NA | 0.00 |
| ***Degradation*** | | |  |  |
| bo8g039420 | BGLU38 \| TGG1 myrosinase (THIOGLUCOSIDE GLUCOHYDROLASE 1); hydrolyzing O-glycosyl compounds / thioglucosidase \| chr5:9079505-9082384 REVERSE | | NA | 1.06 |

NA, not applicable. ^1^Cotyledon values are arranged from highest expression to lowest expression within each functional category using the K-5-8 line. Yellow-highlighted gene expression patterns (but not the genome ID are displayed on the GS biosynthesis and degradation pathways in Figure 4.
