# Supplementary material for: The biochemical composition and transcriptome of cotyledons from Brassica napus lines expressing the AtGL3 transcription factor and exhibiting reduced flea beetle feeding
Source: BMC Plant Biol. 2018 Apr 16;18:64. doi: 10.1186/s12870-018-1277-6 (PMC5902958; doi:10.1186/s12870-018-1277-6)
Supplement: Supplementary file 3 — Figure S1. MAPMAN (heat map) functional overview of changes in gene expression in K-5-8 glabrous cotyledons. MAPMAN (heat map) functional overview of changes in gene expression in glabrous cotyledons in the 10-day-old hairy leaf (K-5-8) B. napus line relative to cv. Westar. The 36 BINs represent MAPMAN sub-cellular function categories. [7497 out of 8037 differentially expressed genes were mapped using this method, with a few genes mapped into more than one category.] The majority of changes involved up-regulated genes. Blue blocks represent individual up-regulated genes. Red blocks represent 29 individual down-regulated genes. The full spectrum of Category 35 genes (unknowns) was too large to fit on the figure. Relative expression intensity scale is in log2, where darkest colour intensity represents log25 and higher/(+ 5) or lower (− 5) relative to Westar. (PPT 484 kb) [file 12870_2018_1277_MOESM3_ESM.ppt]

## Slide 1
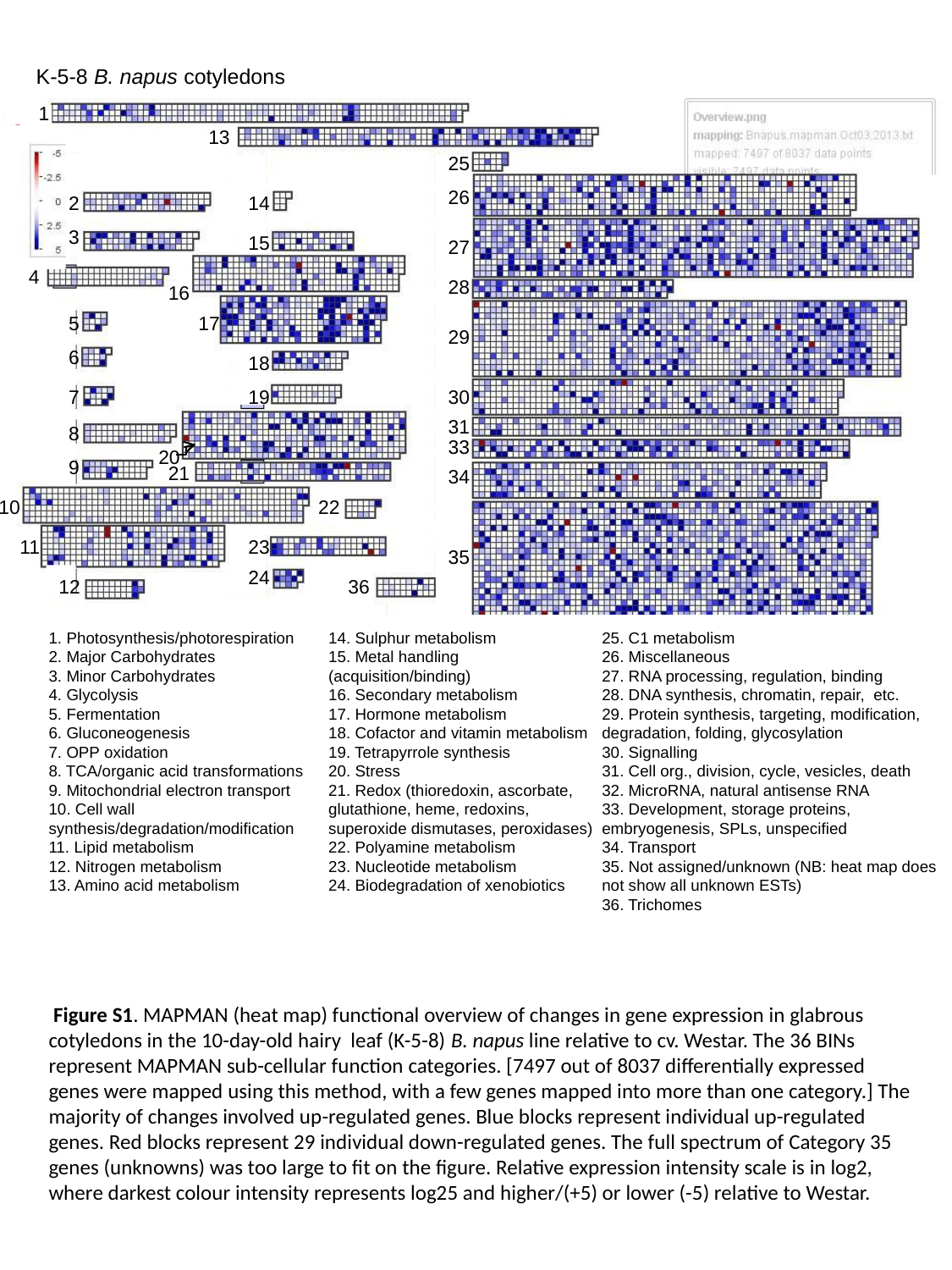

Westar
K-5-8
AtGL3+
K-5-8 B. napus cotyledons
1
13
25
26
2
14
3
15
27
4
28
16
5
17
29
6
18
7
19
30
31
8
33
20
9
21
34
10
22
11
23
35
24
12
36
1. Photosynthesis/photorespiration
2. Major Carbohydrates
3. Minor Carbohydrates
4. Glycolysis
5. Fermentation
6. Gluconeogenesis
7. OPP oxidation
8. TCA/organic acid transformations
9. Mitochondrial electron transport
10. Cell wall synthesis/degradation/modification
11. Lipid metabolism
12. Nitrogen metabolism
13. Amino acid metabolism
14. Sulphur metabolism
15. Metal handling (acquisition/binding)
16. Secondary metabolism
17. Hormone metabolism
18. Cofactor and vitamin metabolism
19. Tetrapyrrole synthesis
20. Stress
21. Redox (thioredoxin, ascorbate, glutathione, heme, redoxins, superoxide dismutases, peroxidases)
22. Polyamine metabolism
23. Nucleotide metabolism
24. Biodegradation of xenobiotics
25. C1 metabolism
26. Miscellaneous
27. RNA processing, regulation, binding
28. DNA synthesis, chromatin, repair, etc.
29. Protein synthesis, targeting, modification, degradation, folding, glycosylation
30. Signalling
31. Cell org., division, cycle, vesicles, death
32. MicroRNA, natural antisense RNA
33. Development, storage proteins, embryogenesis, SPLs, unspecified
34. Transport
35. Not assigned/unknown (NB: heat map does not show all unknown ESTs)
36. Trichomes
 Figure S1. MAPMAN (heat map) functional overview of changes in gene expression in glabrous cotyledons in the 10-day-old hairy leaf (K-5-8) B. napus line relative to cv. Westar. The 36 BINs represent MAPMAN sub-cellular function categories. [7497 out of 8037 differentially expressed genes were mapped using this method, with a few genes mapped into more than one category.] The majority of changes involved up-regulated genes. Blue blocks represent individual up-regulated genes. Red blocks represent 29 individual down-regulated genes. The full spectrum of Category 35 genes (unknowns) was too large to fit on the figure. Relative expression intensity scale is in log2, where darkest colour intensity represents log25 and higher/(+5) or lower (-5) relative to Westar.
