# Supplementary material for: The biochemical composition and transcriptome of cotyledons from Brassica napus lines expressing the AtGL3 transcription factor and exhibiting reduced flea beetle feeding
Source: BMC Plant Biol. 2018 Apr 16;18:64. doi: 10.1186/s12870-018-1277-6 (PMC5902958; doi:10.1186/s12870-018-1277-6)
Supplement: Supplementary file 5 — Figure S3. MAPMAN heat maps of stress responsive genes in glabrous cotyledons. MAPMAN heat maps of stress responsive genes in glabrous cotyledons of (A) 10-day-old hairy leaf (AtGL3+) and (B) ultra hairy leaf (K-5-8) B. napus lines relative to cv. Westar. Blue and red blocks represent individual up- and down-regulated genes. Relative expression intensity scale is in log2 where ±4 represents ± log24 or greater. (PPT 401 kb) [file 12870_2018_1277_MOESM5_ESM.ppt]

## Slide 1
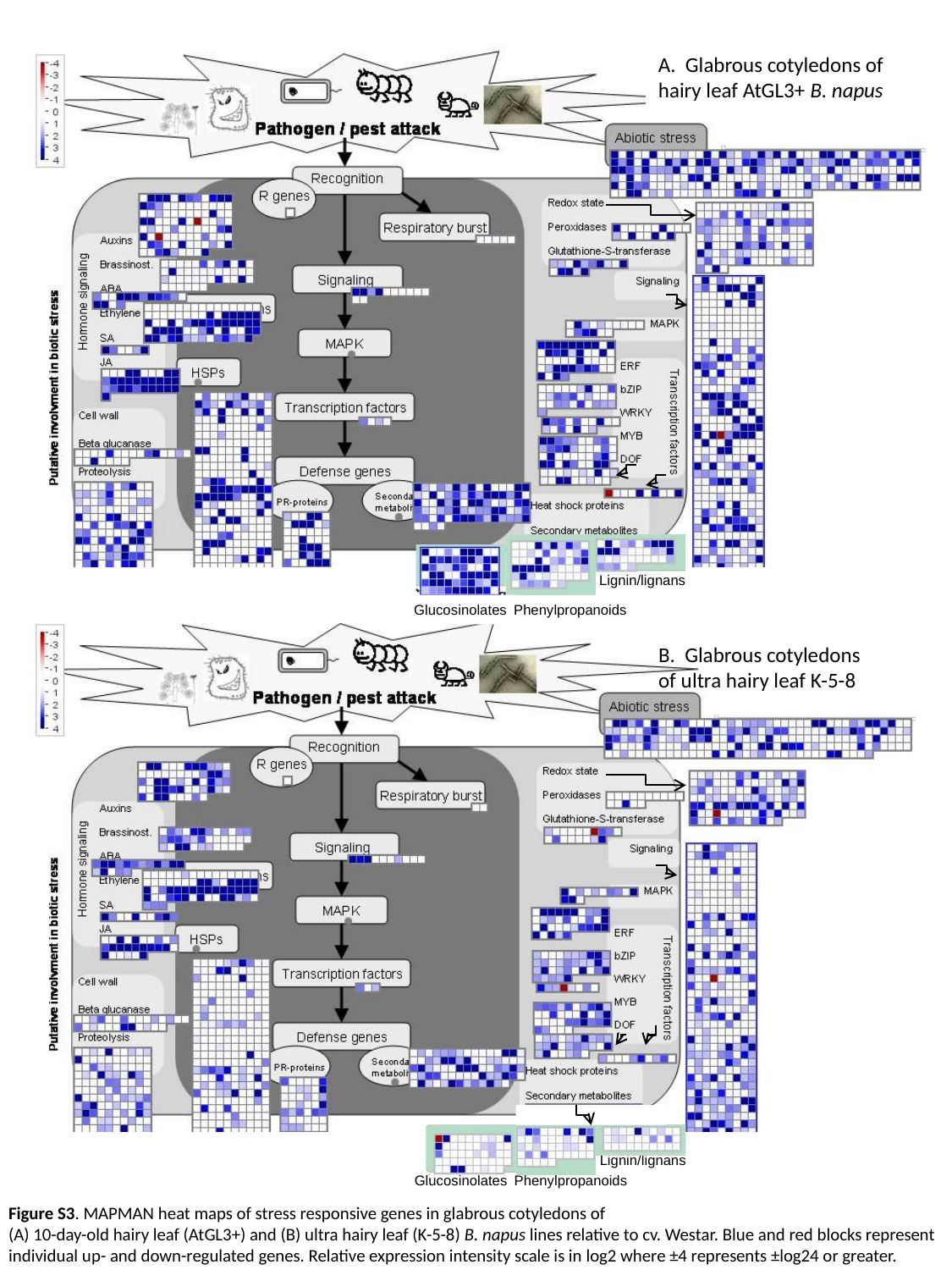

A. Glabrous cotyledons of hairy leaf AtGL3+ B. napus
Lignin/lignans
Glucosinolates
Phenylpropanoids
B. Glabrous cotyledons of ultra hairy leaf K-5-8
Lignin/lignans
Glucosinolates
Phenylpropanoids
Figure S3. MAPMAN heat maps of stress responsive genes in glabrous cotyledons of
(A) 10-day-old hairy leaf (AtGL3+) and (B) ultra hairy leaf (K-5-8) B. napus lines relative to cv. Westar. Blue and red blocks represent individual up- and down-regulated genes. Relative expression intensity scale is in log2 where ±4 represents ±log24 or greater.
