# Supplementary material for: The biochemical composition and transcriptome of cotyledons from Brassica napus lines expressing the AtGL3 transcription factor and exhibiting reduced flea beetle feeding
Source: BMC Plant Biol. 2018 Apr 16;18:64. doi: 10.1186/s12870-018-1277-6 (PMC5902958; doi:10.1186/s12870-018-1277-6)
Supplement: Supplementary file 6 — Figure S4. MAPMAN heat maps of metabolism genes in glabrous cotyledons. MAPMAN heat maps of metabolism genes in glabrous cotyledons of (A) hairy leaf AtGL3+ B. napus and (B) ultra-hairy leaf K-5-8 B. napus, relative to Westar. Maps show numbers of ESTs and expression intensity. Blue blocks represent up-regulated genes. Red blocks represent individual down-regulated genes. Relative expression intensity scale is in log2, where ±5 represents ±log25 or greater. (PPT 287 kb) [file 12870_2018_1277_MOESM6_ESM.ppt]

## Slide 1
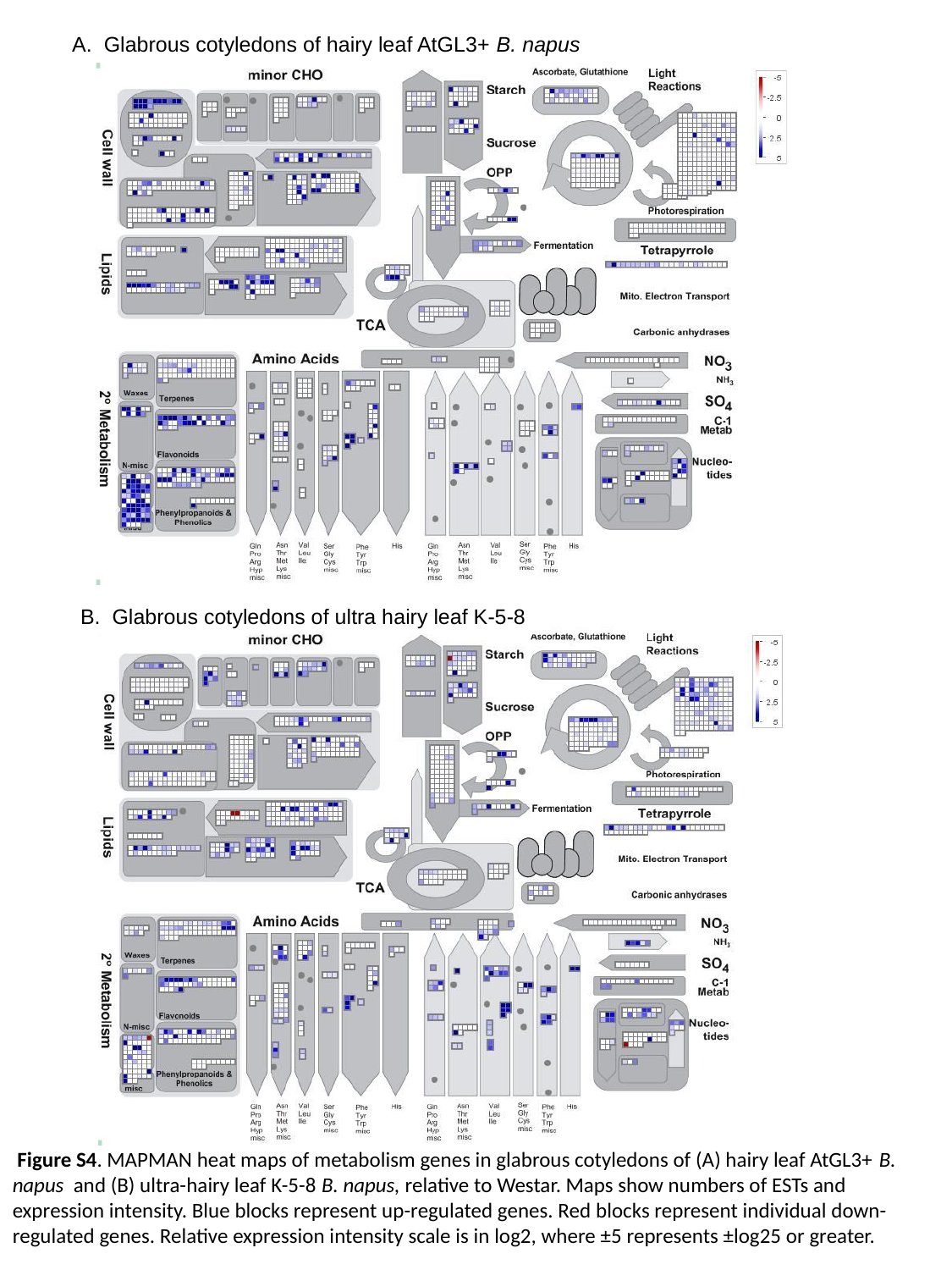

A. Glabrous cotyledons of hairy leaf AtGL3+ B. napus
B. Glabrous cotyledons of ultra hairy leaf K-5-8
 Figure S4. MAPMAN heat maps of metabolism genes in glabrous cotyledons of (A) hairy leaf AtGL3+ B. napus and (B) ultra-hairy leaf K-5-8 B. napus, relative to Westar. Maps show numbers of ESTs and expression intensity. Blue blocks represent up-regulated genes. Red blocks represent individual down-regulated genes. Relative expression intensity scale is in log2, where ±5 represents ±log25 or greater.
