# Supplementary material for: The biochemical composition and transcriptome of cotyledons from Brassica napus lines expressing the AtGL3 transcription factor and exhibiting reduced flea beetle feeding
Source: BMC Plant Biol. 2018 Apr 16;18:64. doi: 10.1186/s12870-018-1277-6 (PMC5902958; doi:10.1186/s12870-018-1277-6)
Supplement: Supplementary file 7 — Figure S5. MAPMAN heat maps of gene regulation and protein-related genes in glabrous cotyledons. MAPMAN heat maps of gene regulation and protein-related genes in glabrous cotyledons of (A) AtGL3+ B. napus and (B) K-5-8 relative to Westar. Maps show numbers of changed ESTs and expression intensity. Blue blocks represent individual up-regulated genes. Red blocks represent individual down-regulated genes. Relative expression intensity scale is in log2 where ±5 represents ±log24 or greater. (PPT 279 kb) [file 12870_2018_1277_MOESM7_ESM.ppt]

## Slide 1
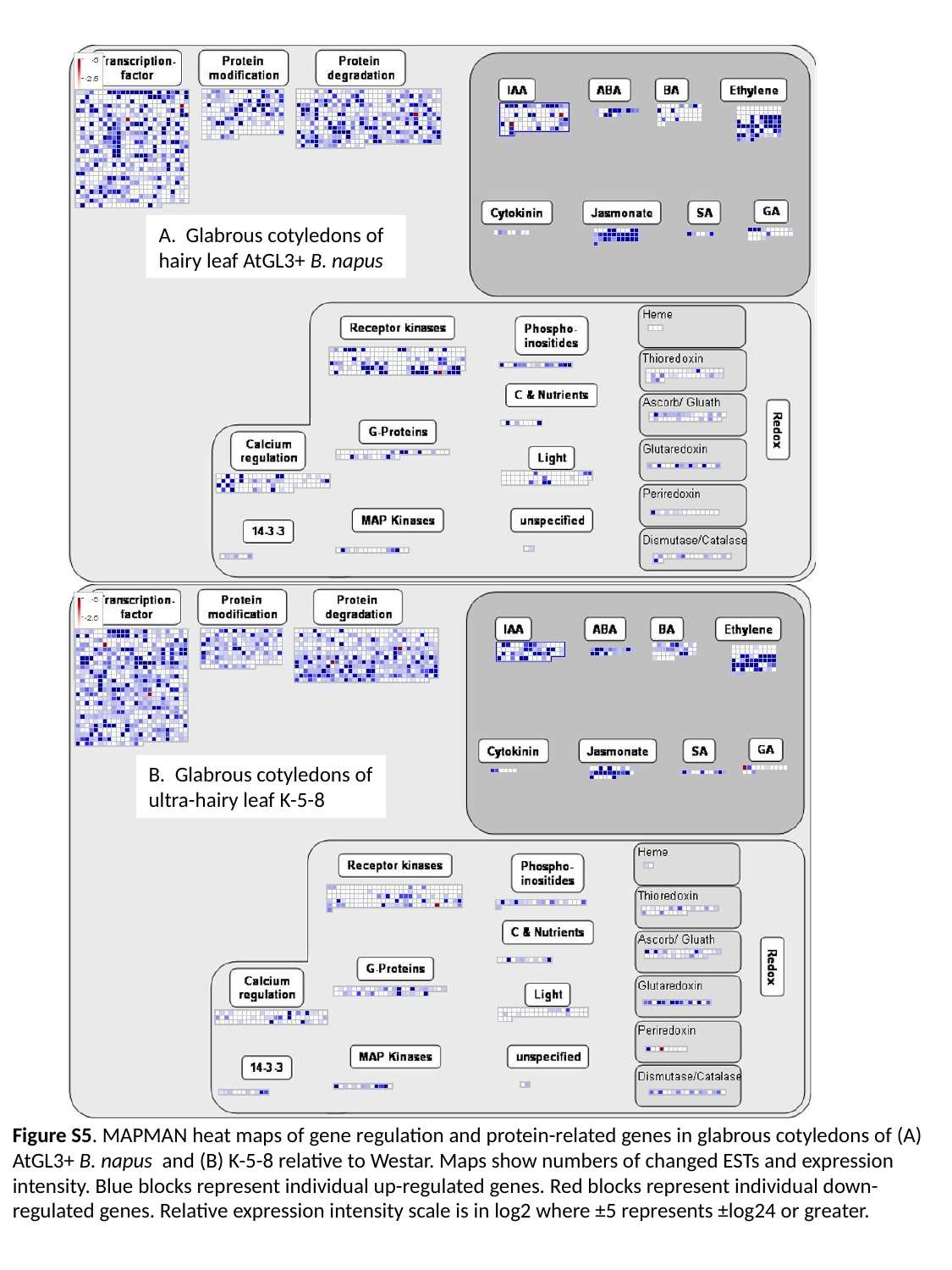

A. Glabrous cotyledons of hairy leaf AtGL3+ B. napus
B. Glabrous cotyledons of ultra-hairy leaf K-5-8
Figure S5. MAPMAN heat maps of gene regulation and protein-related genes in glabrous cotyledons of (A) AtGL3+ B. napus and (B) K-5-8 relative to Westar. Maps show numbers of changed ESTs and expression intensity. Blue blocks represent individual up-regulated genes. Red blocks represent individual down-regulated genes. Relative expression intensity scale is in log2 where ±5 represents ±log24 or greater.
